# Persistent spread of carbapenemase-producing *Klebsiella pneumoniae* in acute care hospitals in 36 European countries (the CCRE survey): a prospective, multicentre, cross-sectional, epidemiological, microbiological, and genomic surveillance study

**DOI:** 10.1016/j.lanmic.2025.101320

**Published:** 2026-06

**Authors:** Inga Fröding, Sophia David, Corin Yeats, Khalil Abu-Dahab, Barbara Albiger, Nabil-Fareed Alikhan, Erik Alm, Sara Byfors, Natacha Couto, Julio Diaz Caballero, Christian G Giske, Erika Matuschek, Marius Linkevicius, Daniel Palm, Olov Svartström, Marc J Struelens, Karin Tegmark Wisell, Dominique L Monnet, Alma Brolund, David M Aanensen, Anke Kohlenberg, EURGen-Net CCRE Survey Working Group, Andi Koraqi, Artan Bego, Petra Apfalter, Rainer Hartl, Te-Din Huang, Katrien Latour, Maja Travar, Stefana Sabtcheva, Arjana Tambić-Andrašević, Jaroslav Hrabák, Helena Žemličková, Panayiota Maikanti Charalambous, Anette M Hammerum, Anastasia Bilozor, Marika Jürna-Ellam, Jari Jalava, Kati Räisänen, Laurent Dortet, Ines Noll, Niels Pfennigwerth, Kyriaki Tryfinopoulou, Alkiviadis Vatopoulos, Ákos Tóth, Kristjan Orri Helgason, Martin Cormican, Giulia Errico, Monica Monaco, Arsim Kurti, Lul Raka, Baiba Niedre-Otomere, Jelena Razmuk, Jekaterina Sinotova, Marie Meo, Monique Perrin, Denise Micallef, Nina Nestorova, Milena Lopičić, Vineta Vuksanović, Daan Notermans, Karuna E W Vendrik, Ana Kaftandzieva, Dugagjin Osmani, Ørjan Samuelsen, Elżbieta Literacka, Manuela Caniça, Vera Manageiro, Irina Codita, Brandusa Lixandru, Ivana Ćirković, Deana Medić, Milan Nikš, Mateja Pirš, Javier E Cañada-García, María Pérez-Vázquez, Petra Edquist, Karin Westmo, Hüsniye Şimşek, Serap Süzük Yildiz, Katie L Hopkins, Danièle Meunier

**Affiliations:** a The Public Health Agency of Sweden, Solna, Sweden; b Department of Laboratory Medicine, Division of Clinical Microbiology, Karolinska Institutet, Stockholm, Sweden; c Centre for Genomic Pathogen Surveillance, Pandemic Sciences Institute, University of Oxford, Oxford, UK; d European Centre for Disease Prevention and Control, Solna, Sweden; e Department of Clinical Microbiology, Karolinska University Hospital, Stockholm, Sweden; f European Committee on Antimicrobial Susceptibility Testing Development Laboratory, Växjö, Sweden; g Faculty of Medicine, Université Libre de Bruxelles, Brussels, Belgium

## Abstract

**Background:**

Carbapenem-resistant Enterobacterales pose a substantial threat to patients and health-care systems. We conducted a survey of carbapenem-resistant and/or colistin-resistant Enterobacterales (CCRE survey) in 37 European countries to describe their occurrence, geographical distribution, and population dynamics and inform control policies. We report the results of *Klebsiella pneumoniae* species complex isolates in this study.

**Methods:**

In this cross-sectional, epidemiological, microbiological, and genomic study conducted in all EU, European Economic Area and EU candidate countries as of 2019, hospital microbiology laboratories were selected on the basis of population coverage. Participating laboratories collected, from patient samples, the first ten successive isolates of carbapenem-resistant or carbapenem-susceptible increased exposure (carbapenem-R/I) *K pneumoniae* species complex or *Escherichia coli*, and carbapenem-susceptible (carbapenem-S) comparator isolates of the same species, accompanied by patient epidemiological and clinical information. Isolate collection started in 2019, with three possible starting dates—ie, March 1, April 1, or May 1, 2019, and ended after collection of ten carbapenem-R/I and carbapenem-S isolates or a maximum period of 6 months. Isolates were tested for phenotypic susceptibility to 16 antimicrobial agents of relevance to *K pneumoniae* species complex. Whole-genome sequencing was performed centrally using Illumina technology. Isolates from the CCRE survey were compared with those from the European Survey of Carbapenemase-Producing Enterobacteriaceae (EuSCAPE) study.

**Findings:**

1566 carbapenem-R/I and 1407 carbapenem-S *K pneumoniae* species complex isolates collected from patients in 302 hospitals in 36 countries (one country did not send isolates) were analysed in this study. The high-risk lineages identified during a previous similar survey in 2013–14 (EuSCAPE) were found to continue to circulate across European hospitals in 2019 (ST11, ST15, ST101, and ST258/512). Moreover, concerning shifts in the pathogen population were observed. First, a higher proportion of carbapenem-R/I isolates was found to carry a carbapenemase gene in the CCRE survey (1398 [89·3%] of 1566) than in EuSCAPE (657 [69·6%] of 944), mainly related to increased acquisition of carbapenemase genes by high-risk lineages. Of note, among ST307 isolates from all hospitals, the proportion of carbapenem-R/I isolates carrying a carbapenemase gene increased from 14 (60·9%) of 23 in EuSCAPE to 164 (91·1%) of 180 in the CCRE survey. Second, an expansion of emerging multidrug-resistant lineages (ST147, ST307, and ST39) was also noted: Among 113 hospitals that contributed *K pneumoniae* species complex isolates to both EuSCAPE and the CCRE survey, the proportion of ST147 increased from 16 (3·4%) of 476 in EuSCAPE to 49 (7·4%) of 662 carbapenem-R/I isolates in the CCRE survey, that of ST307 increased from 15 (3·2%) of 476 to 88 (13·3%) of 662, and that of ST39 increased from 3 (0·6%) of 476 to 10 (1·5%) of 662. Third, there was an increased spread of isolates harbouring acquired virulence loci: isolates with the highest Kleborate virulence score of five increased from 7 (0·4%) of 1717 in EuSCAPE to 40 (1·3%) of 2973 in the CCRE survey. Notably, the increase was mainly observed in the carbapenem-S-group.

**Interpretation:**

The survey findings portray an escalating epidemiological situation and suggest that control measures have not been able to interrupt transmission of high-risk lineages of carbapenemase-producing *K pneumoniae* in European hospitals. The heterogeneous and evolving situation with regards to circulating lineages and dominant carbapenemase genes requires strengthening and continuous adaptation of diagnostic, treatment, and control measures guided by genomic surveillance.

**Funding:**

European Centre for Disease Prevention and Control and Centre for Genomic Pathogen Surveillance.

## Introduction

Carbapenem-resistant Enterobacterales (CRE), including *Klebsiella pneumoniae*, pose a substantial threat to patients and health-care systems worldwide.^1^^,^^2^ Carbapenem-resistant *K pneumoniae* bloodstream infections are associated with high mortality, related to few remaining antimicrobial treatment options.^3^^,^^4^ The spread of high-risk lineages in health-care settings has been identified as a major route of dissemination of carbapenemase genes among *K pneumoniae* in European countries.^5^ *K pneumoniae* infections that are both hypervirulent and multidrug-resistant (MDR) have increasingly been reported, presenting an increased threat to patients and health-care systems.^6^

**Research in context**

**Evidence before this study**

On Oct 4, 2025, we performed a literature search in PubMed with the terms “(carbapenemase−producing)” OR “(carbapenem−resistant)” AND “(*Klebsiella pneumoniae*)” AND “(Europe)” AND “(genomic surveillance)” for reports published between Jan 1, 2000, and Oct 4, 2025, with no language restrictions and no other exclusion criteria. This search identified 129 publications, which mainly consisted of national surveillance studies or surveillance studies in a few countries, outbreak investigations, or single-hospital studies, or analyses of the distribution of specific sequence types (STs) or specific resistance genes. The only other epidemiological and genomic study with similarly comprehensive European coverage and standardised sampling identified was the European Survey of Carbapenemase-Producing Enterobacteriaceae (EuSCAPE), conducted from 2013 to 2014, to which we compare the data generated in this study.

**Added value of this study**

This is the second large-scale structured genomic survey of carbapenem-resistant Enterobacterales, coordinated by the European Centre for Disease Prevention and Control, following the EuSCAPE in 2013–14, largely following the same countries, and partly, the same hospitals after 5 years. We provide epidemiological, microbiological, and whole-genome sequencing data on 2973 carbapenem-resistant or carbapenem-susceptible, increased exposure (carbapenem-R/I) and carbapenem-susceptible *K pneumoniae* species complex isolates collected in 302 hospitals across 36 countries in 2019. We analysed epidemiological characteristics and report phenotypic susceptibility patterns and associated resistance and virulence genes.

An increase was observed in the proportion of carbapenem-R/I *K pneumoniae* isolates carrying carbapenemase genes, compared with that in the EuSCAPE study. We also describe the geographical distribution of *K pneumoniae* high-risk lineages in Europe and their evolution over time, highlighting the continued transmission of previously detected lineages (ST101, ST11, ST15, and ST258/512) and emergence of new high-risk lineages (ST147, ST307, and ST39) in European health-care facilities. Finally, we provide a reference dataset for further studies on *K pneumoniae*, including data from countries where whole-genome sequencing-based national surveillance is not yet established.

**Implications of all the available evidence**

High-risk lineages of carbapenem-resistant *K pneumoniae* have continued to disseminate widely across the European hospital network, showing that applied control measures have been insufficient to stop their spread. In this study, we show that there is not one single epidemic of carbapenem-resistant Enterobacterales, but heterogenous epidemiological situations ranging from sporadic cases to endemicity involving different combinations of carbapenemases and STs in European countries. The findings highlight the need for more effective implementation of infection prevention and control measures and antimicrobial stewardship, in addition to the tailored adaptation of diagnostic, treatment, and control approaches guided by genomic surveillance.

In 2019, we conducted a survey of carbapenem-resistant and/or colistin-resistant Enterobacterales (CCRE survey) by collecting epidemiological, microbiological, and whole-genome sequencing (WGS) data on *K pneumoniae* and *Escherichia coli* across 37 European countries. The CCRE survey is the second large-scale genomic survey on the European level after the European Survey of Carbapenemase-Producing Enterobacteriaceae (EuSCAPE) conducted in 2013–14.^5^^,^^7^ The CCRE survey included capacity building for participating National Reference Laboratories (NRLs), starting with an initial assessment of NRL capacity, surveillance, and containment activities,^8^ followed by training and external quality assessment focused on CRE.

The primary public health objective of the CCRE survey was to evaluate the occurrence, geographical distribution, and population dynamics of CRE high-risk lineages and related transmissible resistance elements in Europe, to inform control measures. In this Article, we present findings focusing on carbapenem-resistant *K pneumoniae*, and highlight key changes in the epidemiology, pathogen population structure, and distribution across acute care hospitals and countries in Europe.

## Methods

### Study design and data

For this cross-sectional study, the CCRE survey protocol was adapted from the EuSCAPE study.^5^^,^^7^ The detailed methods are outlined in the European Centre for Disease Prevention and Control (ECDC) and CCRE survey protocols.^9^, ^10^, ^11^ In short, all EU, European Economic Area and EU candidate countries as of 2019 were invited to participate and the protocol aimed for recruitment of one sentinel hospital per Nomenclature of Territorial Units for Statistics (NUTS)-level-2 region in each country for geographical representativeness. Each recruited hospital microbiology laboratory collected the first ten non-duplicate isolates of carbapenem-resistant or carbapenem-susceptible increased exposure (hereafter called carbapenem-R/I) *K pneumoniae* species complex or *E coli*, based on 2019 European Committee on Antimicrobial Susceptibility Testing (EUCAST) breakpoints,^12^ from clinical or screening samples of consecutive patients. For each registered carbapenem-R/I isolate, the next available carbapenem-susceptible (carbapenem-S) isolate of the same species was collected as a comparator. Isolate collection started in 2019 with three possible starting dates (ie, March 1, April 1, or May 1, 2019) and ended after collection of ten carbapenem-R/I and carbapenem-S isolates or a maximum period of 6 months. Epidemiological data, as outlined in the protocol,^9^ were reported via an online form to a central database. All data were pseudonymised and collected in accordance with the European Parliament and Council decisions on the epidemiological surveillance and control of communicable disease in the European community. Ethics approval and informed consent were, thus, not required.

The main differences in the CCRE survey protocol, as compared with the EuSCAPE protocol,^5^^,^^7^ were the inclusion of isolates from screening samples (as compared with only clinical samples in EuSCAPE), geographical coverage based on NUTS-2 regions, and addition of ceftazidime–avibactam to the phenotypic antimicrobial susceptibility testing (AST) panel.

All species within the *K pneumoniae* species complex were accepted (ie, species confirmed as *K pneumoniae*, *Klebsiella quasipneumoniae*, *Klebsiella variicola*, *Klebsiella quasivariicola*, or *Klebsiella africana*). Inclusion required testing of a minimum of one carbapenem among ertapenem, imipenem, or meropenem. EUCAST breakpoint table (version 9), 2019, was used for interpretation of AST results.^12^ Isolates were included as carbapenem-R/I when one or more of the tested carbapenems were in the resistant (R) or susceptible, increased exposure (I) categories and as carbapenem-S when results were in the susceptible (S) category for all tested carbapenems.

### Procedures

Each participating laboratory performed AST using established routine methods, primarily disc diffusion or broth microdilution, according to EUCAST guidelines.^12^ Results of gradient tests and commercial automated systems were also accepted. The recommended AST panel included 16 antimicrobial agents relevant to the *K pneumoniae* species complex: amoxicillin–clavulanic acid, aztreonam, piperacillin–tazobactam, cefotaxime, ceftazidime, cefepime, ceftazidime–avibactam, ertapenem, imipenem, meropenem, ciprofloxacin, amikacin, gentamicin, tobramycin, colistin, and trimethoprim–sulphamethoxazole. Results were interpreted by the participating laboratories, with the exception of the AST results for carbapenems, for which central validation of the susceptible, standard dosing regimen; susceptible, increased exposure; and resistant (SIR) categorisation was performed. For colistin, only results reported as tested with broth microdilution were included in the analysis.

### Whole-genome sequencing

WGS was performed using Illumina NovaSeq 6000 (Illumina Inc, San Diego, CA, USA) instruments with 150-base pair paired-end reads. Raw reads were assembled using SPAdes (version 3.10.0) with the “careful” flag provided and the “−cov-cutoff” flag set to “auto”.^13^ Genome assemblies were annotated using Prokka (version 1.5).^14^ Species designations and assembly metrics were obtained from genome assemblies using Kleborate (version 2.1.0).^15^ Isolates with species designations that were inconsistent with the species identification provided by the NRLs (other than inconsistencies within the *K pneumoniae* species complex itself), and those with 1000 contigs or more or an assembly length outside of 4·5–6·5 Mb, were excluded. Kleborate was also used to identify the presence of resistance and virulence genes. Inconsistencies regarding carbapenemase genes between NRL and WGS results that could not be resolved after a clarification request to the NRL led to exclusion. New allele numbers for novel putative carbapenemase variants were assigned by submission to GenBank.

To construct phylogenetic trees, core gene sets were identified using Panaroo (version 1.2.9) in the “moderate” mode.^16^ A bespoke alignment was generated for all *K pneumoniae* species complex genomes containing 3642 genes present in 95% or more of isolates from each of the four identified species and used to build a tree with Mashtree (version 2.3).^17^ A separate core gene alignment was generated for isolates of the *K pneumoniae* species containing 4198 genes present in 95% or more of the genomes and used to build a tree with RAxML-NG (version 1.0.3).^18^ Multilocus sequence typing and core genome multilocus sequence typing profiles, in addition to life identification number codes, were obtained using BIGSdb-Pasteur, with new types defined wherever necessary. Replicon typing data were obtained using Pathogenwatch (version 12.2.1)^19^ using the PlasmidFinder database (version 2021-11-29).^20^ The *K pneumoniae* species complex isolates from the CCRE survey (2973 isolates from 302 hospitals in 36 countries) were compared with those from the EuSCAPE study (1717 isolates from 244 hospitals in 32 countries).^5^ Microreact was used for visualisation of phylogenetic trees with metadata.^21^

### Outcomes

The main outcomes of this study were the distribution of STs and resistance and virulence genes of carbapenem-R/I *K pneumoniae* circulating in European acute care hospitals in 36 countries in comparison to that in carbapenem-S comparator isolates from the same hospitals. Epidemiological characteristics and phenotypic AST results of both groups were also evaluated. Comparison of the data from this study to those from the previous EuSCAPE project allowed detection of changes in the composition of STs and resistance and virulence genes over time.

### Statistical analysis

Statistical analyses were performed using RStudio (version 4.3.1). For proportions shown in table 1 and appendix 1 p 1, robust standard errors clustered by hospital and country were estimated with the sandwich estimator, and 95% logit-transformed Wald confidence intervals were obtained.

**Table 1:** Epidemiological characteristics associated with carbapenem-R/I and carbapenem-S *Klebsiella pneumoniae* species complex isolates and the most frequent sequence types from the CCRE survey

	*K pneumoniae* species complex									
	Total n (%, 95% CI)	**Carbapenem-R/I *K pneumoniae* species complex (n=1566)**								Carbapenem-S *K pneumoniae* species complex (n=1407); n (%, 95% CI)
		Total n (%, 95% CI)	ST258/ST512 n (%, 95% CI)	ST101 n (%, 95% CI)	ST11 n (%, 95% CI)	ST307 n (%, 95% CI)	ST147 n (%, 95% CI)	ST15 n (%, 95% CI)	Other STs n (%, 95% CI)	
Countries	36	36	18	20	22	21	20	16	34	34
Hospitals	302	295	71	85	68	79	51	41	225	274
Isolates	2973	1566	232	209	187	180	95	81	582	1407
**Sex**										
Male	1553 (52·2%, 50·4–54·0)	903 (57·7%, 55·2–60·1)	139 (59·9%, 53·5–66·0)	134 (64·1%, 57·4–70·3)	101 (54·0%, 46·8–61·0)	101 (56·1%, 48·8–63·2)	54 (56·8%, 46·7–66·5)	42 (51·9%, 41·0–62·5)	332 (57·0%, 53·0–61·0)	650 (46·2%, 43·6–48·8)
Female	1322 (44·5%, 42·7–46·3)	619 (39·5%, 37·1–42·0)	86 (37·1%, 31·1–43·5)	66 (31·6%, 25·6–38·2)	83 (44·4%, 37·4–51·6)	76 (42·2%, 35·2–49·6)	38 (40·0%, 30·6–50·2)	36 (44·4%, 34·0–55·4)	234 (40·2%, 36·3–44·2)	703 (50·0%, 47·4–52·6)
Missing	98 (3·3%, 2·7–4·0)	44 (2·8%, 2·1–3·8)	7 (3·0%, 1·4–6·2)	9 (4·3%, 2·3–8·1)	3 (1·6%, 0·5–4·9)	3 (1·7%, 0·5–5·1)	3 (3·2%, 1·0–9·4)	3 (3·7%, 1·2–10·9)	16 (2·7%, 1·7–4·4)	54 (3·8%, 3·0–5·0)
**Age**										
0–19 years	157 (5·3%, 4·5–6·1)	57 (3·6%, 2·8–4·7)	3 (1·3%, 0·4–3·9)	7 (3·3%, 1·6–6·9)	2 (1·1%, 0·3–4·2)	2 (1·1%, 0·3–4·3)	2 (2·1%, 0·5–8·1)	0	41 (7·0%, 5·2–9·4)	100 (7·1%, 5·9–8·6)
20–39 years	218 (7·3%, 6·4–8·3)	106 (6·8%, 5·6–8·1)	21 (9·1%, 6·0–13·5)	18 (8·6%, 5·5–13·3)	12 (6·4%, 3·7–11·0)	6 (3·3%, 1·5–7·2)	6 (6·3%, 2·9–13·4)	5 (6·2%, 2·6–14·1)	38 (6·5%, 4·8–8·8)	112 (8·0%, 6·7–9·5)
40–59 years	517 (17·4%, 16·1–18·8)	283 (18·1%, 16·2–20·1)	42 (18·1%, 13·7–23·6)	43 (20·6%, 15·6–26·6)	31 (16·6%, 11·9–22·6)	31 (17·2%, 12·4–23·5)	22 (23·2%, 15·7–32·7)	9 (11·1%, 5·9–20·1)	105 (18·0%, 15·1–21·4)	234 (16·6%, 14·8–18·7)
60–79 years	1332 (44·8%, 43·0–46·6)	733 (46·8%, 44·3–49·3)	112 (48·3%, 41·9–54·7)	93 (44·5%, 37·9–51·3)	93 (49·7%, 42·6–56·9)	89 (49·4%, 42·2–56·7)	42 (44·2%, 34·5–54·3)	40 (49·4%, 38·6–60·2)	264 (45·4%, 41·4–49·4)	599 (42·6%, 40·0–45·2)
≥80 years	638 (21·5%, 20·0–23·0)	343 (21·9%, 19·9–24·0)	47 (20·3%, 15·6–25·9)	38 (18·2%, 13·5–24·0)	46 (24·6%, 18·9–31·3)	50 (27·8%, 21·7–34·8)	20 (21·1%, 14·0–30·5)	25 (30·9%, 21·7–41·8)	117 (20·1%, 17·0–23·6)	295 (21·0%, 18·9–23·2)
Missing	111 (3·7%, 3·1–4·5)	44 (2·8%, 2·1–3·8)	7 (3·0%, 1·4–6·2)	10 (4·8%, 2·6–8·7)	3 (1·6%, 0·5–4·9)	2 (1·1%, 0·3–4·3)	3 (3·2%, 1·0–9·4)	2 (2·5%, 0·6–9·4)	17 (2·9%, 1·8–4·6)	67 (4·8%, 3·8–6·0)
**Hospitalisation status**										
Outpatient	559 (18·8%, 17·4–20·2)	215 (13·7%, 12·1–15·5)	30 (12·9%, 9·2–17·9)	19 (9·1%, 5·9–13·8)	25 (13·4%, 9·2–19·1)	18 (10·0%, 6·4–15·3)	15 (15·8%, 9·7–24·6)	26 (32·1%, 22·8–43·0)	82 (14·1%, 11·5–17·2)	344 (24·4%, 22·3–26·8)
Inpatient	2300 (77·4%, 75·8–78·8)	1291 (82·4%, 80·5–84·2)	193 (83·2%, 77·8–87·5)	180 (86·1%, 80·7–90·2)	154 (82·4%, 76·2–87·2)	160 (88·9%, 83·4–92·7)	75 (78·9%, 69·5–86·0)	51 (63·0%, 51·9–72·8)	478 (82·1%, 78·8–85·0)	1009 (71·7%, 69·3–74·0)
Missing	114 (3·8%, 3·2–4·6)	60 (3·8%, 3·0–4·9)	9 (3·9%, 2·0–7·3)	10 (4·8%, 2·6–8·7)	8 (4·3%, 2·1–8·3)	2 (1·1%, 0·3–4·3)	5 (5·3%, 2·2–12·1)	4 (4·9%, 1·9–12·5)	22 (3·8%, 2·5–5·7)	54 (3·8%, 3·0–5·0)
**Type of ward**										
Medical	1257 (42·3%, 40·5–44·1)	624 (39·8%, 37·4–42·3)	93 (40·1%, 34·0–46·5)	70 (33·5%, 27·4–40·2)	73 (39·0%, 32·3–46·2)	84 (46·7%, 39·5–54·0)	36 (37·9%, 28·7–48·1)	31 (38·3%, 28·3–49·3)	237 (40·7%, 36·8–44·8)	633 (45·0%, 42·4–47·6)
Intensive care	637 (21·4%, 20·0–22·9)	436 (27·8%, 25·7–30·1)	60 (25·9%, 20·6–31·9)	72 (34·4%, 28·3–41·2)	53 (28·3%, 22·3–35·2)	51 (28·3%, 22·2–35·4)	26 (27·4%, 19·3–37·2)	11 (13·6%, 7·7–23·0)	163 (28·0%, 24·5–31·8)	201 (14·3%, 12·6–16·2)
Surgical	452 (15·2%, 14·0–16·5)	239 (15·3%, 13·6–17·1)	40 (17·2%, 12·9–22·7)	42 (20·1%, 15·2–26·1)	36 (19·3%, 14·2–25·6)	27 (15·0%, 10·5–21·0)	12 (12·6%, 7·3–21·0)	7 (8·6%, 4·2–17·1)	75 (12·9%, 10·4–15·9)	213 (15·1%, 13·4–17·1)
Other	450 (15·1%, 13·9–16·5)	183 (11·7%, 10·2–13·4)	22 (9·5%, 6·3–14·0)	12 (5·7%, 3·3–9·8)	20 (10·7%, 7·0–16·0)	10 (5·6%, 3·0–10·0)	15 (15·8%, 9·7–24·6)	28 (34·6%, 25·0–45·6)	76 (13·1%, 10·6–16·1)	267 (19·0%, 17·0–21·1)
Missing	177 (6·0%, 5·2–6·9)	84 (5·4%, 4·4–6·6)	17 (7·3%, 4·6–11·5)	13 (6·2%, 3·6–10·4)	5 (2·7%, 1·1–6·3)	8 (4·4%, 2·2–8·6)	6 (6·3%, 2·9–13·4)	4 (4·9%, 1·9–12·5)	31 (5·3%, 3·8–7·5)	93 (6·6%, 5·4–8·0)
**Infection or carriage status**										
Carriage	496 (16·7%, 15·4–18·1)	313 (20·0%, 18·1–22·0)	37 (15·9%, 11·8–21·3)	24 (11·5%, 7·8–16·6)	50 (26·7%, 20·9–33·6)	31 (17·2%, 12·4–23·5)	17 (17·9%, 11·4–27·0)	17 (21·0%, 13·4–31·3)	137 (23·5%, 20·3–27·2)	183 (13·0%, 11·3–14·9)
Infection	2135 (71·8%, 70·2–73·4)	1087 (69·4%, 67·1–71·6)	160 (69·0%, 62·7–74·6)	163 (78·0%, 71·9–83·1)	131 (70·1%, 63·1–76·2)	124 (68·9%, 61·7–75·2)	71 (74·7%, 65·0–82·5)	61 (75·3%, 64·7–83·5)	377 (64·8%, 60·8–68·6)	1048 (74·5%, 72·1–76·7)
Undetermined	243 (8·2%, 7·2–9·2)	119 (7·6%, 6·4–9·0)	25 (10·8%, 7·4–15·5)	13 (6·2%, 3·6–10·4)	4 (2·1%, 0·8–5·6)	19 (10·6%, 6·8–16·0)	5 (5·3%, 2·2–12·1)	1 (1·2%, 0·2–8·3)	52 (8·9%, 6·9–11·5)	124 (8·8%, 7·4–10·4)
Missing	99 (3·3%, 2·7–4·0)	47 (3·0%, 2·3–4·0)	10 (4·3%, 2·3–7·8)	9 (4·3%, 2·3–8·1)	2 (1·1%, 0·3–4·2)	6 (3·3%, 1·5–7·2)	2 (2·1%, 0·5–8·1)	2 (2·5%, 0·6–9·4)	16 (2·7%, 1·7–4·4)	52 (3·7%, 2·8–4·8)
**Organ system or location of infection or carriage**										
Urinary tract	1252 (42·1%, 40·3–43·9)	598 (38·2%, 35·8–40·6)	80 (34·5%, 28·6–40·8)	71 (34·0%, 27·9–40·7)	91 (48·7%, 41·6–55·8)	60 (33·3%, 26·8–40·6)	39 (41·1%, 31·6–51·2)	37 (45·7%, 35·1–56·6)	220 (37·8%, 33·9–41·8)	654 (46·5%, 43·9–49·1)
Lower respiratory tract	363 (12·2%, 11·1–13·4)	203 (13·0%, 11·4–14·7)	37 (15·9%, 11·8–21·3)	35 (16·7%, 12·3–22·4)	27 (14·4%, 10·1–20·3)	25 (13·9%, 9·6–19·8)	12 (12·6%, 7·3–21·0)	6 (7·4%, 3·3–15·6)	61 (10·5%, 8·2–13·2)	160 (11·4%, 9·8–13·1)
Intra-abdominal	130 (4·4%, 3·7–5·2)	78 (5·0%, 4·0–6·2)	15 (6·5%, 3·9–10·5)	10 (4·8%, 2·6–8·7)	7 (3·7%, 1·8–7·7)	9 (5·0%, 2·6–9·3)	5 (5·3%, 2·2–12·1)	2 (2·5%, 0·6–9·4)	30 (5·2%, 3·6–7·3)	52 (3·7%, 2·8–4·8)
Blood stream	439 (14·8%, 13·5–16·1)	231 (14·8%, 13·1–16·6)	46 (19·8%, 15·2–25·5)	44 (21·1%, 16·0–27·1)	17 (9·1%, 5·7–14·2)	43 (23·9%, 18·2–30·7)	10 (10·5%, 5·7–18·5)	6 (7·4%, 3·3–15·6)	65 (11·2%, 8·9–14·0)	208 (14·8%, 13·0–16·7)
Skin or soft tissue	228 (7·7%, 6·8–8·7)	131 (8·4%, 7·1–9·8)	21 (9·1%, 6·0–13·5)	21 (10·0%, 6·6–14·9)	14 (7·5%, 4·5–12·3)	15 (8·3%, 5·1–13·4)	10 (10·5%, 5·7–18·5)	8 (9·9%, 5·0–18·6)	42 (7·2%, 5·4–9·6)	97 (6·9%, 5·7–8·3)
Other	217 (7·3%, 6·4–8·3)	138 (8·8%, 7·5–10·3)	17 (7·3%, 4·6–11·5)	16 (7·7%, 4·7–12·1)	15 (8·0%, 4·9–12·9)	14 (7·8%, 4·7–12·7)	7 (7·4%, 3·5–14·7)	6 (7·4%, 3·3–15·6)	63 (10·8%, 8·5–13·6)	79 (5·6%, 4·5–6·9)
Missing	344 (11·6%, 10·5–12·8)	187 (11·9%, 10·4–13·6)	16 (6·9%, 4·3–11·0)	12 (5·7%, 3·3–9·8)	16 (8·6%, 5·3–13·5)	14 (7·8%, 4·7–12·7)	12 (12·6%, 7·3–21·0)	16 (19·8%, 12·4–29·9)	101 (17·4%, 14·5–20·7)	157 (11·2%, 9·6–12·9)
**Type of sample**										
Clinical sample	2718 (91·4%, 90·4–92·4)	1380 (88·1%, 86·4–89·6)	219 (94·4%, 90·6–96·7)	190 (90·9%, 86·2–94·1)	162 (86·6%, 80·9–90·8)	168 (93·3%, 88·6–96·2)	89 (93·7%, 86·6–97·1)	72 (88·9%, 79·9–94·1)	480 (82·5%, 79·2–85·4)	1338 (95·1%, 93·8–96·1)
Screening sample	245 (8·2%, 7·3–9·3)	183 (11·7%, 10·2–13·4)	13 (5·6%, 3·3–9·4)	17 (8·1%, 5·1–12·7)	25 (13·4%, 9·2–19·1)	12 (6·7%, 3·8–11·4)	6 (6·3%, 2·9–13·4)	9 (11·1%, 5·9–20·1)	101 (17·4%, 14·5–20·7)	62 (4·4%, 3·4–5·6)
Missing	10 (0·3%, 0·2–0·6)	3 (0·2%, 0·1–0·6)	0	2 (1·0%, 0·2–3·8)	0	0	0	0	1 (0·2%, 0·0–1·2)	7 (0·5%, 0·2–1·0)
**Origin of sample**										
Urine	1348 (45·3%, 43·6–47·1)	646 (41·3%, 38·8–43·7)	85 (36·6%, 30·7–43·0)	71 (34·0%, 27·9–40·7)	82 (43·9%, 36·9–51·1)	65 (36·1%, 29·4–43·4)	47 (49·5%, 39·5–59·5)	44 (54·3%, 43·4–64·9)	252 (43·3%, 39·3–47·4)	702 (49·9%, 47·3–52·5)
Blood	517 (17·4%, 16·1–18·8)	270 (17·2%, 15·4–19·2)	49 (21·1%, 16·3–26·9)	48 (23·0%, 17·8–29·2)	18 (9·6%, 6·1–14·8)	45 (25·0%, 19·2–31·9)	12 (12·6%, 7·3–21·0)	8 (9·9%, 5·0–18·6)	90 (15·5%, 12·7–18·6)	247 (17·6%, 15·7–19·6)
Lower respiratory tract	356 (12·0%, 10·9–13·2)	200 (12·8%, 11·2–14·5)	39 (16·8%, 12·5–22·2)	33 (15·8%, 11·4–21·4)	25 (13·4%, 9·2–19·1)	24 (13·3%, 9·1–19·1)	12 (12·6%, 7·3–21·0)	6 (7·4%, 3·3–15·6)	61 (10·5%, 8·2–13·2)	156 (11·1%, 9·5–12·8)
Wound swabs	196 (6·6%, 5·8–7·5)	103 (6·6%, 5·5–7·9)	20 (8·6%, 5·6–13·0)	14 (6·7%, 4·0–11·0)	10 (5·3%, 2·9–9·7)	10 (5·6%, 3·0–10·0)	8 (8·4%, 4·3–16·0)	5 (6·2%, 2·6–14·1)	36 (6·2%, 4·5–8·5)	93 (6·6%, 5·4–8·0)
Aspirates	88 (3·0%, 2·4–3·6)	41 (2·6%, 1·9–3·5)	9 (3·9%, 2·0–7·3)	8 (3·8%, 1·9–7·5)	1 (0·5%, 0·1–3·7)	6 (3·3%, 1·5–7·2)	2 (2·1%, 0·5–8·1)	1 (1·2%, 0·2–8·3)	14 (2·4%, 1·4–4·0)	47 (3·3%, 2·5–4·4)
Soft tissue samples	56 (1·9%, 1·5–2·4)	36 (2·3%, 1·7–3·2)	9 (3·9%, 2·0–7·3)	6 (2·9%, 1·3–6·3)	3 (1·6%, 0·5–4·9)	6 (3·3%, 1·5–7·2)	2 (2·1%, 0·5–8·1)	6 (7·4%, 3·3–15·6)	4 (0·7%, 0·3–1·8)	20 (1·4%, 0·9–2·2)
Catheter exit site	52 (1·7%, 1·3–2·3)	35 (2·2%, 1·6–3·1)	5 (2·2%, 0·9–5·1)	3 (1·4%, 0·5–4·4)	14 (7·5%, 4·5–12·3)	3 (1·7%, 0·5–5·1)	0	2 (2·5%, 0·6–9·4)	8 (1·4%, 0·7–2·7)	17 (1·2%, 0·8–1·9)
Reproductive tract samples	14 (0·5%, 0·3–0·8)	1 (0·1%, 0·0–0·5)	0	0	0	0	0	0	1 (0·2%, 0·0–1·2)	13 (0·9%, 0·5–1·6)
Bone and joint specimens	10 (0·3%, 0·2–0·6)	8 (0·5%, 0·3–1·0)	1 (0·4%, 0·1–3·0)	2 (1·0%, 0·2–3·8)	2 (1·1%, 0·3–4·2)	0	2 (2·1%, 0·5–8·1)	0	1 (0·2%, 0·0–1·2)	2 (0·1%, 0·0–0·6)
Cerebrospinal fluid	2 (0·1%, 0·0–0·3)	1 (0·1%, 0·0–0·5)	1 (0·4%, 0·1–3·0)	0	0	0	0	0	0	1 (0·1%, 0·0–0·5)
Other	292 (9·8%, 8·8–10·9)	195 (12·5%, 10·9–14·2)	12 (5·2%, 3·0–8·9)	22 (10·5%, 7·0–15·5)	30 (16·0, % 11·4–22·0)	18 (10·0%, 6·4–15·3)	8 (8·4%, 4·3–16·0)	9 (11·1%, 5·9–20·1)	96 (16·5%, 13·7–19·7)	97 (6·9%, 5·7–8·3)
Missing	42 (1·4%, 1·0–1·9)	30 (1·9%, 1·3–2·7)	2 (0·9%, 0·2–3·4)	2 (1·0%, 0·2–3·8)	2 (1·1%, 0·3–4·2)	3 (1·7%, 0·5–5·1)	2 (2·1%, 0·5–8·1)	0	19 (3·3%, 2·1–5·1)	12 (0·9%, 0·5–1·5)
**Hospital or community acquisition**										
Community-onset	1018 (34·2%, 32·6–36·0)	359 (22·9%, 20·9–25·1)	45 (19·4%, 14·8–25·0)	38 (18·2%, 13·5–24·0)	58 (31·0%, 24·8–38·0)	32 (17·8%, 12·8–24·1)	27 (28·4%, 20·2–38·3)	33 (40·7%, 30·6–51·8)	126 (21·6%, 18·5–25·2)	659 (46·8%, 44·2–49·5)
Hospital-acquired	1512 (50·9%, 49·1–52·7)	971 (62·0%, 59·6–64·4)	155 (66·8%, 60·5–72·6)	140 (67·0%, 60·3–73·0)	110 (58·8%, 51·6–65·7)	130 (72·2%, 65·2–78·3)	55 (57·9%, 47·7–67·4)	38 (46·9%, 36·3–57·8)	343 (58·9%, 54·9–62·9)	541 (38·5%, 35·9–41·0)
Missing	443 (14·9%, 13·7–16·2)	236 (15·1%, 13·4–16·9)	32 (13·8%, 9·9–18·9)	31 (14·8%, 10·6–20·3)	19 (10·2%, 6·6–15·4)	18 (10·0%, 6·4–15·3)	13 (13·7%, 8·1–22·2)	10 (12·3%, 6·7–21·5)	113 (19·4%, 16·4–22·8)	207 (14·7%, 13·0–16·7)
**Previous hospital admission within 6 months**∗										
Yes	1193 (40·1%, 38·4–41·9)	741 (47·3%, 44·9–49·8)	129 (55·6%, 49·1–61·9)	89 (42·6%, 36·0–49·4)	95 (50·8%, 43·7–57·9)	90 (50·0%, 42·7–57·3)	43 (45·3%, 35·5–55·4)	37 (45·7%, 35·1–56·6)	258 (44·3%, 40·3–48·4)	452 (32·1%, 29·7–34·6)
No	857 (28·8%, 27·2–30·5)	343 (21·9%, 19·9–24·0)	39 (16·8%, 12·5–22·2)	46 (22·0%, 16·9–28·1)	52 (27·8%, 21·8–34·7)	33 (18·3%, 13·3–24·7)	22 (23·2%, 15·7–32·7)	17 (21·0%, 13·4–31·3)	134 (23·0%, 19·8–26·6)	514 (36·5%, 34·1–39·1)
Missing	923 (31·0%, 29·4–32·7)	482 (30·8%, 28·5–33·1)	64 (27·6%, 22·2–33·7)	74 (35·4%, 29·2–42·1)	40 (21·4%, 16·1–27·9)	57 (31·7%, 25·3–38·8)	30 (31·6%, 23·0–41·6)	27 (33·3%, 23·9–44·3)	190 (32·6%, 29·0–36·6)	441 (31·3%, 29·0–33·8)
**Previous residence in a long-term care or residential care facility within 6 months**										
Yes	189 (6·4%, 5·5–7·3)	133 (8·5%, 7·2–10·0)	28 (12·1%, 8·5–16·9)	10 (4·8%, 2·6–8·7)	19 (10·2%, 6·6–15·4)	20 (11·1%, 7·3–16·6)	9 (9·5%, 5·0–17·3)	11 (13·6%, 7·7–23·0)	36 (6·2%, 4·5–8·5)	56 (4·0%, 3·1–5·1)
No	1487 (50·0%, 48·2–51·8)	731 (46·7%, 44·2–49·2)	125 (53·9%, 47·4–60·2)	97 (46·4%, 39·7–53·2)	103 (55·1%, 47·9–62·1)	67 (37·2%, 30·5–44·5)	43 (45·3%, 35·5–55·4)	35 (43·2%, 32·8–54·2)	261 (44·8%, 40·8–48·9)	756 (53·7%, 51·1–56·3)
Missing	1297 (43·6%, 41·9–45·4)	702 (44·8%, 42·4–47·3)	79 (34·1%, 28·2–40·4)	102 (48·8%, 42·1–55·6)	65 (34·8%, 28·3–41·9)	93 (51·7%, 44·4–58·9)	43 (45·3%, 35·5–55·4)	35 (43·2%, 32·8–54·2)	285 (49·0%, 44·9–53·0)	595 (42·3%, 39·7–44·9)
**Previous travel within 6 months**†										
Yes	79 (2·7%, 2·1–3·3)	66 (4·2%, 3·3–5·3)	8 (3·4%, 1·7–6·8)	13 (6·2%, 3·6–10·4)	6 (3·2%, 1·4–7·0)	6 (3·3%, 1·5–7·2)	3 (3·2%, 1·0–9·4)	4 (4·9%, 1·9–12·5)	26 (4·5%, 3·1–6·5)	13 (0·9%, 0·5–1·6)
No reported travel or information not available	2894 (97·3%, 96·7–97·9)	1500 (95·8%, 94·7–96·7)	224 (96·6%, 93·2–98·3)	196 (93·8%, 89·6–96·4)	181 (96·8%, 93·0–98·6)	174 (96·7%, 92·8–98·5)	92 (96·8%, 90·6–99·0)	77 (95·1%, 87·5–98·1)	556 (95·5%, 93·5–96·9)	1394 (99·1%, 98·4–99·5)

CCRE=carbapenem-resistant and/or colistin-resistant Enterobacterales. I=susceptible, increased exposure. R=resistant. S=susceptible. ST=sequence type.

∗ Previous hospital admission within 6 months includes direct transfer from another hospital.

† Previous travel within 6 months includes direct transfer from a hospital in another country and previous hospital admission within 6 months in another country.

### Role of the funding source

The ECDC staffmanaged European Antimicrobial Resistance Genes Surveillance Network (EURGen-Net) and provided input to the study design, data collection, data analysis, data interpretation, and writing of the report. The Centre for Genomic Pathogen Surveillance funded the WGS analysis and provided input to the study design, data collection, data analysis, data interpretation, and writing of the report. The decision to submit for publication was jointly taken by representatives of the ECDC, Centre for Genomic Pathogen Surveillance, and The Public Health Agency of Sweden.

## Results

National technical coordinators in 37 European countries recruited 527 hospitals for the CCRE survey, of which 346 (65·7%) registered 3349 *K pneumoniae* species complex isolates during the sampling period in 2019. Registered isolates were filtered during various validation and quality control steps, obtaining 2973 isolates in the survey dataset (appendix 1 p 3). In this study, we describe the epidemiological, phenotypic, and genomic analyses of 2973 *K pneumoniae* species complex isolates (1566 carbapenem-R/I and 1407 carbapenem-S); these isolates originated from 302 hospitals in 36 countries (Albania registered eight *K pneumoniae* species complex isolates but did not transfer them to the central strain collection) and fulfilled the inclusion and quality control criteria (appendix 2). The quality control criteria included viable growth, correct species identification, and consistent SIR categorisation with registered carbapenem-S or carbapenem-R/I-group. Included isolates were from 1553 (52·2%) male and 1322 (44·5%) female patients and from patients in the following age groups: 0–19 years, 157 (5·3%) isolates; 20–39 years, 218 (7·3%) isolates; 40–59 years, 517 (17·4%) isolates; 60–79 years, 1332 (44·8) isolates; and ≥80 years, 638 (21·5%) isolates (table 1). The number of *K pneumoniae* species complex isolates contributed per country was highly variable, ranging from two (Iceland and Kosovo) to 509 (Italy) isolates (table 2). Of the 327 NUTS-2 regions in participating countries, 227 (69·4%) were covered by at least one *K pneumoniae* species complex isolate in the final dataset.

**Table 2:** Carbapenem-R/I and carbapenem-S *Klebsiella pneumoniae* species complex isolates and carbapenemase genes by country, collected and analysed during the CCRE survey

	Hospitals	Carbapenem-R/I *K pneumoniae* species complex isolates (n=1566)								Carbapenem-S *K pneumoniae* species complex isolates	Total *K pneumoniae* species complex isolates
		Total carbapenem-R/I *K pneumoniae* species complex isolates	***K pneumoniae* species complex isolates carrying carbapenemase genes**						Isolates without carbapenemase genes		
			Isolates carrying carbapenemase genes	Isolates carrying *bla*_KPC_	Isolates carrying *bla*_NDM_	Isolates carrying *bla*_OXA-48−_like	Isolates carrying *bla*_VIM_	Isolates carrying other or multiple carbapenemase genes			
Albania	0	0	0	0	0	0	0	0	0	0	0
Austria	7	19	17	9	1	5	1	1	2	17	36
Belgium	14	51	49	5	6	36	0	2	2	49	100
Bosnia and Herzegovina	1	10	10	0	0	10	0	0	0	7	17
Bulgaria	10	84	77	42	33	1	0	1	7	84	168
Croatia	9	59	56	14	2	40	0	0	3	60	119
Cyprus	1	8	8	7	0	0	1	0	0	9	17
Czechia	8	27	14	3	7	3	1	0	13	28	55
Denmark	3	3	3	0	2	1	0	0	0	0	3
Estonia	1	2	0	0	0	0	0	0	2	3	5
Finland	5	11	11	7	2	1	0	1	0	0	11
France	12	40	21	2	6	12	0	1	19	48	88
Germany	18	33	26	8	4	12	1	1	7	35	68
Greece	15	128	128	75	31	2	12	8	0	126	254
Hungary	8	40	32	1	10	5	16	0	8	25	65
Iceland	1	1	1	0	0	1	0	0	0	1	2
Ireland	6	8	6	1	2	3	0	0	2	14	22
Italy	32	278	274	248	9	4	10	3	4	231	509
Kosovo	1	1	0	0	0	0	0	0	1	1	2
Latvia	1	3	1	1	0	0	0	0	2	3	6
Lithuania	1	8	8	8	0	0	0	0	0	8	16
Luxembourg	2	3	3	2	0	1	0	0	0	3	6
Malta	1	9	9	0	2	6	0	1	0	10	19
Montenegro	1	10	2	0	0	2	0	0	8	8	18
Netherlands	13	23	18	1	6	8	0	3	5	21	44
North Macedonia	1	3	3	0	2	1	0	0	0	2	5
Norway	3	7	6	3	2	1	0	0	1	7	14
Poland	12	55	45	2	41	1	1	0	10	58	113
Portugal	7	52	49	42	0	7	0	0	3	33	85
Romania	9	66	62	20	7	30	0	5	4	51	117
Serbia	9	69	61	7	11	43	0	0	8	38	107
Slovakia	3	30	30	10	20	0	0	0	0	24	54
Slovenia	7	24	13	3	0	10	0	0	11	23	47
Spain	24	176	160	34	4	112	8	2	16	181	357
Sweden	7	20	17	3	6	6	0	2	3	19	39
Türkiye	25	138	130	26	19	69	0	16	8	123	261
UK	24	67	48	16	6	20	0	6	19	57	124
All countries	302	1566	1398	600	241	453	51	53	168	1407	2973

Data are n. The CCRE survey was conducted in 37 countries. Albania did not transfer any isolates to the central strain collection and was excluded, resulting in a final dataset from 36 countries. CCRE=carbapenem-resistant and/or colistin-resistant Enterobacterales. I=susceptible, increased exposure. R=resistant. S=susceptible.

Most *K pneumoniae* species complex isolates were associated with infections; the most frequent organ system of infection or colonisation was the urinary tract, followed by the bloodstream and respiratory tract for both carbapenem-R/I and carbapenem-S isolates (table 1). More carbapenem-R/I (436 [27·8%, 95% CI 25·7–30·1] of 1566 isolates) than carbapenem-S isolates (201 [14·3%, 12·6–16·2] of 1407 isolates) were associated with patients in intensive care units. In addition, 971 (62·0%, 59·6–64·4) carbapenem-R/I isolates were classified as hospital-acquired, as compared with 541 (38·5%, 35·9–41·0) carbapenem-S isolates. A previous hospital admission within 6 months before the sampling date was documented for 741 (47·3%, 44·9–49·8) patients with carbapenem-R/I and 452 (32·1%, 29·7–34·6) patients with carbapenem-S isolates (table 1).

**Figure 1:**
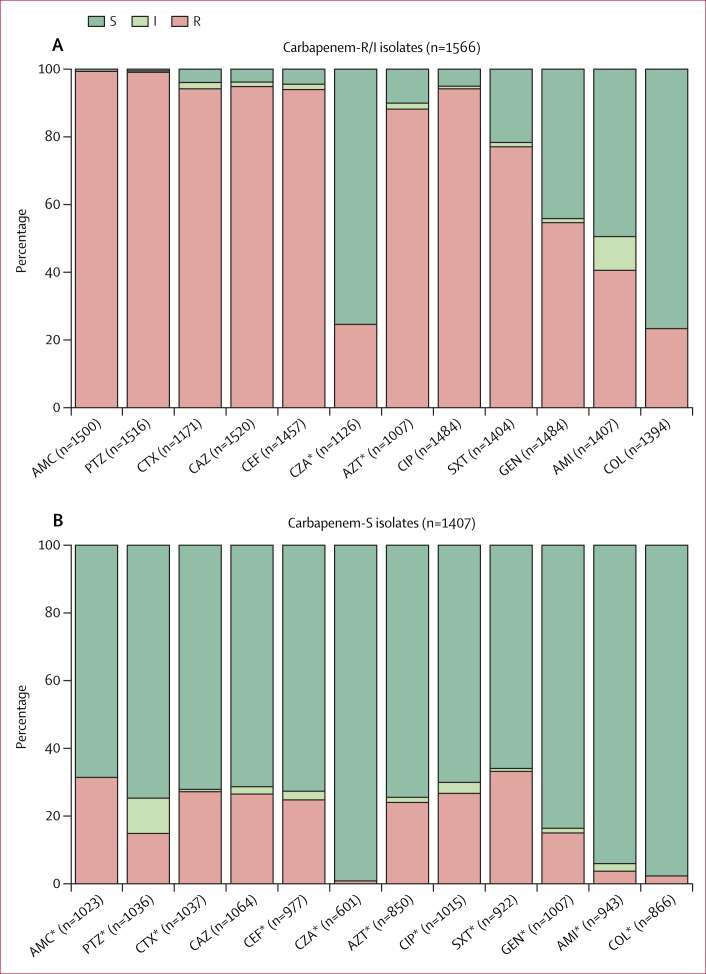
Phenotypic susceptibility for select antimicrobial agents of *Klebsiella pneumoniae* species complex isolates from the CCRE survey (A) 1566 carbapenem-R/I and (B) 1407 carbapenem-S *Klebsiella pneumoniae* species complex isolates. The numbers of tested isolates are shown in parentheses for each antimicrobial agent. Antimicrobial agents with AST results available for less than 75% of isolates are marked with an asterisk. Note: The susceptibility interpretation shown is according to reported AST, performed and interpreted by the NRLs, according to EUCAST breakpoint table version 9.0, 2019.^12^ Graphs show the proportion of S, R, and I of the tested isolates. The number of isolates with AST results varies between antimicrobial agents. For some antimicrobials, breakpoints have been changed in subsequent years. For piperacillin–tazobactam, isolates categorised as I in 2019, would be categorised as R from version 11.0, 2021. For colistin, breakpoints are in brackets since the EUCAST breakpoint table version 12.0, 2022. For isolates categorised as colistin S in the graphs, combination with another active agent or measure would be required. For aminoglycosides, breakpoints for monotherapy are restricted to infections originating from the urinary tract, and isolates categorised as I for gentamicin or amikacin in 2019, would be categorised as R since EUCAST breakpoint table version 10.0, 2020. AMC=amoxicillin–clavulanic acid. AMI=amikacin. AST=antimicrobial susceptibility testing. AZT=aztreonam. CAZ=ceftazidime. CCRE=carbapenem-resistant and/or colistin-resistant Enterobacterales. CEF=cefepime. CIP=ciprofloxacin. COL=colistin. CTX=cefotaxime. CZA=ceftazidime–avibactam. EUCAST=European Committee on Antimicrobial Susceptibility Testing. GEN=gentamicin. I=susceptible, increased exposure. NRLs=National Reference Laboratories. PTZ=piperacillin–tazobactam. R=resistant. S=susceptible. SXT=trimethoprim–sulphamethoxazole.

More than 75% of isolates were tested for susceptibility to amoxicillin–clavulanic acid, piperacillin–tazobactam, cefotaxime, ceftazidime, cefepime, ciprofloxacin, trimethoprim–sulphamethoxazole, gentamicin, amikacin, and colistin (appendix 1 p 4). Carbapenem-R/I *K pneumoniae* species complex isolates showed high levels of resistance to other antimicrobial agents such as ciprofloxacin and gentamicin (figure 1A, appendix 1 p 2). Colistin and ceftazidime–avibactam were the two agents with the lowest proportion of carbapenem-R/I *K pneumoniae* species complex isolates testing resistant (figure 1A, appendix 1 p 2). Colistin resistance was observed in 312 (24·7%, 17·6–33·4) of 1264 tested carbapenemase-producing isolates, comparable to the 186 (28·7%) observed in EuSCAPE (648 tested isolates).^7^ Clinically relevant proportions of carbapenem-S isolates resistant to key agents were also observed (figure 1B, appendix 1 p 2). MDR, defined as R or I to at least one agent from three or more antimicrobial categories,^22^ was observed in 1531 (97·8%, 90·7–99·5) of 1566 carbapenem-R/I and 367 (26·1%, 17·1–37·7) of 1407 carbapenem-S *K pneumoniae* species complex isolates.

**Figure 2:**
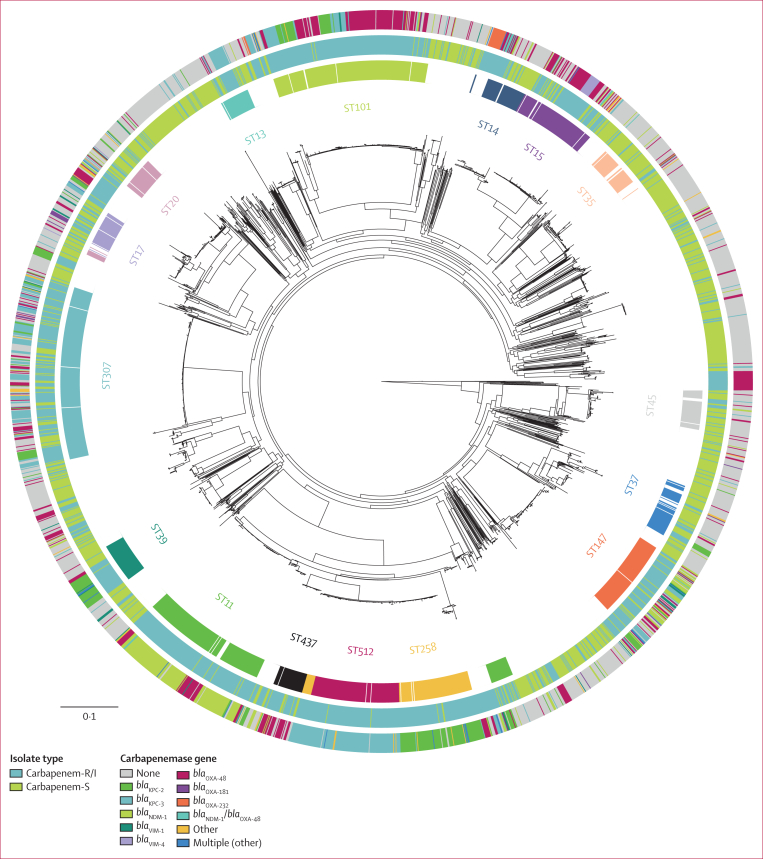
Phylogenetic tree of 2834 *Klebsiella pneumoniae* isolates from the CCRE survey The most frequently observed STs have been highlighted on the tree, and the metadata rings show the isolate type (carbapenem-R/I or carbapenem−S) and the carbapenemase gene. The scale bar represents the number of SNPs per variable site. An interactive version of the tree with additional metadata and genotyping data is available at Microreact.org. CCRE=carbapenem-resistant and/or colistin-resistant Enterobacterales. I=susceptible, increased exposure. R=resistant. S=susceptible. SNPs=single nucleotide polymorphisms. STs=sequence types.

Genomic analysis revealed that the 2973 *K pneumoniae* species complex isolates were from four phylogenetically distinct species and included 2834 isolates of *K pneumoniae*, 50 of *K quasipneumoniae,* 88 of *K variicola*, and one of *K quasivariicola* (appendix 1 p 5). A phylogenetic tree of the *K pneumoniae* isolates showed high diversity, albeit with the carbapenem-R/I isolates largely concentrated into several lineages (figure 2). Across the four species, sequence types (STs) could be unambiguously identified for 2922 (98·3%) of 2973 isolates and included 53 novel ones.

The carbapenem-R/I isolates belonged to 152 STs, of which, based on previously described definitions,^23^ the dominant (>5% of isolates) STs were ST258/512, ST101, ST11, ST307, ST147, and ST15 (table 1, figure 2). These STs form distinct lineages in the phylogenetic tree, with STs 258 and 512 (which are single-locus variants) grouped together as they form a single lineage with no substantial evolutionary separation between them.^5^ The six dominant STs were geographically widespread across participating European countries, having each been isolated from between 41 and 85 hospitals in 16–22 countries (table 1). However, their relative prevalence varied substantially by country (appendix 1 p 6). Hospital and country clusters of these STs are numerous in the phylogenetic tree, and individual countries often had multiple independent introductions of each ST. The carbapenem-S isolates were more diverse than carbapenem-R/I isolates and belonged to 478 STs, all of which were found at an isolate frequency of less than 5%.

We identified one or more carbapenemase genes (or novel related variants) in 1398 (89·3%) of 1566 carbapenem-R/I isolates. 23 known carbapenemase gene variants were detected, in addition to six novel variants with high similarity to known carbapenemase gene variants (appendix 1 p 7). However, four gene variants dominated, with 1265 (90·5%) of 1398 carbapenemase-positive isolates carrying one or more of *bla*_OXA−48_ (435 [31·1%]), *bla*_KPC−3_ (347 [24·8%]), *bla*_NDM−1_ (263 [18·8%]), or *bla*_KPC−2_ (250 [17·9%]), or a combination of these. 52 isolates (3·7%) carried two carbapenemase genes, and the most common combination was *bla*_NDM−1_/*bla*_OXA−48_, which was detected in 27 isolates. Ten (0·7%) of 1407 carbapenem-S isolates carried a single carbapenemase gene, including six isolates with *bla*_OXA−48_, three isolates with *bla*_KPC−2_, and one isolate with *bla*_VIM−1_.

The prevalence of different carbapenemase genes varied by country (table 2, appendix 1 p 8). Most carbapenemase variants were distributed widely across different lineages of the *K pneumoniae* species complex, although we also found specific associations with dominant STs, including of *bla*_KPC−2_ with ST258, *bla*_KPC−3_ with ST307 and ST512, *bla*_NDM−1_ with ST11, *bla*_VIM−4_ with ST15, and *bla*_OXA−48_ with ST101 (figure 2).

**Figure 3:**
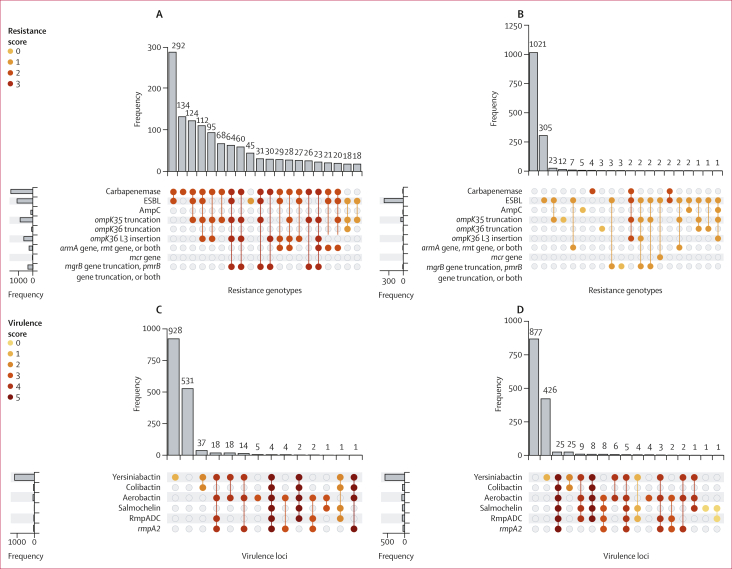
Combinations of resistance genes or mutations and virulence loci among *Klebsiella pneumoniae* species complex isolates Combinations of resistance genes or mutations among (A) carbapenem-R/I and (B) carbapenem-S *K pneumoniae* species complex isolates. Virulence loci among (C) carbapenem-R/I and (D) carbapenem-S *K pneumoniae* species complex isolates. Frequencies of the top 20 combinations (A, B) or all the combinations (C, D) are shown in the upper bar plot, whereas the frequencies of individual loci are shown on the left. The colour scales marking individual combinations of loci represent the resistance and virulence scores defined by Kleborate. I=susceptible, increased exposure. R=resistant. S=susceptible.

We found a strong co-occurrence of resistance mechanisms among carbapenem-R/I isolates, as compared with that among carbapenem-S isolates (figure 3A, B). For example, 1003 (64·0%) of 1566 carbapenem-R/I isolates carried an extended-spectrum β-lactamase gene as compared with 357 (25·4%) of 1407 carbapenem-S isolates. 911 (58·2%) carbapenem-R/I isolates possessed one or more porin mutations (*ompK35* or *ompK36* truncation, or an *ompK36* loop 3 insertion), all of which affect susceptibility to β-lactam antibiotics, as compared with 59 (4·2%) of 1407 carbapenem-S isolates. The presence of metallo-β-lactamase-genes explained most of the phenotypic ceftazidime–avibactam resistance detected in 277 (24·6%) of the 1126 tested carbapenem-R/I isolates. However, 38 (7·5%) of the 508 tested isolates with *bla*_KPC_ and 9 (3·2%) of the 283 tested isolates with *bla*__OXA-48−__like genes were also resistant to ceftazidime–avibactam.

Furthermore, 324 (20·7%) of the 1566 carbapenem-R/I isolates possessed an *mgrB*/*pmrB* truncation, associated with colistin resistance, compared with 12 (0·9%) of the 1407 carbapenem-S isolates. 11 isolates with *mcr* gene variants (*mcr-8* and *mcr-9* and other inexactly matched variants) were detected, but of the eight of them tested with broth microdilution, only one was resistant to colistin. The numbers of isolates with either one of the aminoglycoside resistance genes *armA* or *rmt*, or both these genes, were: 221 (14·1%) of 1566 carbapenem-R/I isolates and nine (0·6%) of 1407 carbapenem-S isolates. We also investigated the presence of alternative carbapenem resistance determinants among carbapenem-R/I isolates that did not have a carbapenemase gene. 101 (60·5%) of 167 isolates had an extended-spectrum β-lactamase gene or AmpC gene, or both, in combination with one or more porin mutations, which could potentially explain the carbapenem-R/I phenotype.

Acquired virulence loci were typically rare (<6%) in both the groups, with the exception of yersiniabactin, which was found in 1023 (65·3%) of 1566 carbapenem-R/I isolates and 514 (36·5%) of 1407 carbapenem-S isolates. Seven (0·4%) of 1566 carbapenem-R/I isolates and 33 (2·3%) of 1407 carbapenem-S isolates had the highest predicted virulence (virulence score of five) due to simultaneous carriage of the yersiniabactin, colibactin, and aerobactin loci (figure 3C, D). These 40 isolates were from *K pneumoniae* ST23 (19 isolates), ST380 (eight), ST65 (four), and ST268 (three), with all other isolates (six) belonging to single STs. The 19 ST23 isolates, all of which possessed KL1, were obtained from 15 hospitals in 11 countries, whereas the eight ST380 isolates were collected from six hospitals in five countries. Five of the seven carbapenem-R/I isolates with a virulence score of five carried a carbapenemase gene (*bla*_KPC−3_ or *bla*_OXA−48_).

We compared the *K pneumoniae* species complex isolates from the CCRE survey (2973 isolates from 302 hospitals in 36 countries) with those from the EuSCAPE study (1717 isolates from 244 hospitals in 32 countries).^5^ Although the recruited hospitals varied in each survey, 113 hospitals contributed *K pneumoniae* species complex isolates to both surveys. In the longitudinal analysis using only data on carbapenem-R/I isolates (662 for CCRE and 476 for EuSCAPE) from these 113 hospitals, several of the STs (ST11, ST15, ST101, and ST258/512) that were dominant among carbapenem-R/I isolates in EuSCAPE were still dominant in the CCRE survey (appendix 1 p 9). However, increases in the proportions of ST147, ST307, and ST39 were also observed within these hospitals between the surveys. The proportions of isolates increased from 16 (3·4%) of 476 in EuSCAPE to 49 (7·4%) of 662 in the CCRE survey for ST147, from 15 (3·2%) of 476 to 88 (13·3%) of 662 for ST307, and from 3 (0·6%) of 476 to 10 (1·5%) of 662 for ST39. In addition, among ST307 isolates from all hospitals, there was an increase in the proportion of carbapenem-R/I isolates carrying a carbapenemase gene from 14 (60·9%) of 23 in EuSCAPE to 164 (91·1%) of 180 in the CCRE survey. A slight increase was also visible for ST101, whereas the proportions of ST11, ST15, and ST258/512 decreased (appendix 1 p 9).

Across all hospitals, a higher proportion of carbapenem-R/I isolates was found to carry a carbapenemase gene in the CCRE survey (1398 [89·3%] of 1566) than in EuSCAPE (657 [69·6%] of 944). For the isolates from shared hospitals, differences were found in the proportions of the major carbapenemase variants among carbapenemase-positive isolates, with increases being observed for *bla*_KPC−2_, *bla*_NDM−1_, and *bla*_OXA−48_, and a decrease for *bla*_KPC−3_ from EuSCAPE to the CCRE survey (appendix 1 p 9).

An increase in ST39 isolates associated with an extensive profile of resistance genes was also noted between the surveys (figure 2). 56 ST39 isolates were identified in the CCRE survey, in contrast, to only eight ST39 isolates from EuSCAPE. Most of the carbapenem-R/I ST39 isolates from the CCRE survey were recovered from Greece. These isolates typically coharboured various resistance loci, with all possessing at least one carbapenemase gene (mainly *bla*_KPC−2_), and most also carrying porin mutations and an *mgrB* truncation. Finally, an increase in the total number of isolates with the highest predicted virulence (score of five) was also detected, from seven (0·4%) of 1717 in EuSCAPE to 40 (1·3%) of 2973 in the CCRE survey. However, this increase was mainly observed in the carbapenem-S group.

## Discussion

We report the epidemiological, microbiological, and genomic characteristics of 2973 *K pneumoniae* species complex isolates obtained from 36 countries across Europe during the CCRE survey in 2019. These results provide an updated view of the ongoing epidemic of carbapenem-resistant *K pneumoniae*, which was previously shown to be driven primarily by the spread of high-risk lineages via hospital networks.^5^ Our findings show that despite urgent calls for public health action, many of the same high-risk lineages identified in 2013–14 via EuSCAPE^5^ have continued to disseminate widely within European health-care systems. However, we also detected concerning changes within the *K pneumoniae* population in the sampled hospitals, including the emergence and expansion of new MDR lineages, an increased acquisition of carbapenemase genes by high-risk lineages, and an increased spread of carbapenem-S isolates harbouring acquired virulence loci. Taken together, these findings indicate a deteriorating epidemiological situation in Europe, mirroring the increases in mortality reported in studies on disease burden^24^ and suggesting that control measures have been insufficient for curbing this epidemic.

The continuing widespread distribution of dominant high-risk clones of *K pneumoniae* is exemplified by ST15, ST147, ST11, ST258/512, ST307, and ST101, which were detected in 16–22 countries in 41–85 hospitals. Several of these STs—ie, ST101, ST11, ST15, and ST258/512, were also dominant among carbapenem-R/I isolates from EuSCAPE (2013–14), showing their persistence over time. However, other STs, including ST147, and especially, ST307, increased in abundance and became new dominant lineages in the European hospitals included in the study by 2019. Both are high-risk lineages that have emerged recently and have spread globally within a short timeframe, probably in part due to their association with the *bla*_CTX−M−15_ gene and other resistance loci.^25^^,^^26^

Comparison of data from the CCRE survey and EuSCAPE also enabled the detection of another MDR clone, ST39, at an earlier stage of its expansion, highlighting the potential for structured survey approaches to direct public health action. A follow-up survey conducted in 2022 confirmed the continued circulation of *K pneumoniae* ST39 within Greek hospitals and prompted an alert for enhanced detection, surveillance, and infection prevention and control measures.^27^ Notably, ST39 was previously implicated in hospital outbreaks in African countries.^28^^,^^29^

The proportion of carbapenem-R/I *K pneumoniae* isolates that carried a carbapenemase gene was considerably higher in the samples from 2019 than in the samples from 2013 to 2014. Although changes in sampling and testing procedures might have contributed to the higher proportions, our finding could also reflect true increased acquisition of carbapenemase genes by high-risk lineages of *K pneumoniae* and dissemination of these clones.

The emergence and spread of *K pneumoniae* strains that are both MDR and hypervirulent is an increasing concern globally. Although previously, such strains were only rarely observed in Europe, sustained health care-associated spread of hypervirulent *K pneumoniae* ST23 carrying carbapenemase genes has now been reported in Ireland.^6^^,^^30^ In the CCRE survey, we detected 40 *K pneumoniae* isolates with virulence loci potentially associated with a hypervirulent phenotype (yersiniabactin, colibactin, and aerobactin) across 33 hospitals in 17 European countries, albeit mainly among carbapenem-S isolates. That hypervirulent *K pneumoniae* isolates might be overlooked in settings where screening and further investigation are targeted at carbapenem-resistant *K pneumoniae* remains a concern, which could lead to potentially undetected spread of hypervirulent *K pneumoniae* lineages.

The strength of the CCRE survey lies in the harmonised sampling and data collection from 36 European countries. However, more than 600 registered isolates, comprising both *K pneumoniae* species complex and *E coli*, were excluded for various reasons. Completeness of the epidemiological and microbiological data varied, and the geographical distribution of isolates was uneven. Inclusion was based on EUCAST clinical breakpoints, which might have resulted in underdetection of carbapenemase genes frequently associated with low carbapenem minimal inhibitory concentrations such as *bla*_OXA-48−_like. Further limitations are the delay in publication of the CCRE survey data and the absence of AST results for antimicrobial substances that received marketing authorisation after the survey, such as meropenem–vaborbactam, cefiderocol, and aztreonam–avibactam. Although both EuSCAPE and the CCRE survey were mainly designed for monitoring high-level trends at the European level, the representativeness of the final datasets is low and cannot be generalised to all hospitals or the whole population of health-care recipients. Importantly, they cannot replace national genomic surveillance due to the low number of included hospitals and isolates.

In conclusion, the pooled WGS and epidemiological data from 36 countries in the CCRE survey enabled the detection of emerging resistant lineages at the European level and highlight heterogenous national epidemiological situations involving a varying mix of STs and carbapenemase genes. The continued emergence and expansion of high-risk lineages suggest that control measures have not been implemented effectively enough to curb transmission. Therefore, we support an urgent focus on proven measures based on infection prevention and control and antimicrobial stewardship, with further delays in their effective implementation most likely to lead to escalating morbidity, mortality, and economic costs and increasing difficulties in controlling the epidemic.

## Data sharing

The raw reads of isolates from this study were deposited in the European Nucleotide Archive under accession numbers PRJEB39943 and PRJEB51224. Isolate assemblies are available from Pathogenwatch collections of *K pneumoniae*, *K quasipneumoniae*, *K variicola*, and *K quasivariicola*. Interactive dashboards containing a phylogeny and metadata are also available via Microreact. The metadata made available under the above links and appendix 2 with publication include all accession numbers, isolate identifiers, sampling dates, country and NUTS-2 region the isolate is originating from, associated patient data for the type of specimen (screening or clinical), the sample site for clinical samples (aspirate, blood, bone and joint specimens, catheter exit site, cerebrospinal fluid, lower respiratory tract specimens, reproductive tract samples, soft tissue samples, urine, wound swab or other), the carbapenem used for classification of isolates (ertapenem, imipenem, or meropenem), related minimum inhibitory concentrations or zone diameters and interpretation according to EUCAST breakpoints, as well as outputs from Kleborate for STs and resistance and virulence genes. The study protocols are available on the ECDC webpage (references 9-11), and the analysis plan will be shared upon request.

## Declaration of interests

IF, SB, and AB declare funding from European Centre for Disease Prevention and Control (ECDC) through framework contract ECDC/2017/021. EM is part of the European Committee on Antimicrobial Susceptibility Testing (EUCAST) Executive and General Committees as Head of the EUCAST Development Laboratory and Co-chair of the Clinical and Laboratory Standards Institute (CLSI)-EUCAST joint working group. KTW has been a National Microbiology Focal Point (2014–21), a Board Member (2021–24) for ECDC, and a Member of the Steering Group on Health Promotion, Disease Prevention, and Management of Non-Communicable Diseases (2022–23) and of the Expert Group on Public Health (2023–24) of the European Commission (Directorate-General for Health and Food Safety [DG SANTE]). DMA declares funding by the National Institute for Health and Care Research, UK, as grant to the University of Oxford (grant number NIHR133307) and by the Wellcome Sanger Institute as core funding to the Centre for Genomic Pathogen Surveillance. All other authors declare no competing interests.

## References

[1] European Centre for Disease Prevention and Control (Feb 3, 2025). Carbapenem-resistant Enterobacterales–third update. https://www.ecdc.europa.eu/en/publications-data/carbapenem-resistant-enterobacterales-rapid-risk-assessment-third-update.

[2] WHO (Sept 4, 2017). Prioritization of pathogens to guide discovery, research and development of new antibiotics for drug-resistant bacterial infections, including tuberculosis. https://www.who.int/publications/i/item/WHO-EMP-IAU-2017.12.

[3] Xu L, Sun X, Ma X (2017). Systematic review and meta-analysis of mortality of patients infected with carbapenem-resistant *Klebsiella pneumoniae*. Ann Clin Microbiol Antimicrob.

[4] Di Pilato V, Henrici De Angelis L, Aiezza N (2022). Resistome and virulome accretion in an NDM-1-producing ST147 sublineage of *Klebsiella pneumoniae* associated with an outbreak in Tuscany, Italy: a genotypic and phenotypic characterisation. Lancet Microbe.

[5] David S, Reuter S, Harris SR (2019). Epidemic of carbapenem-resistant *Klebsiella pneumoniae* in Europe is driven by nosocomial spread. Nat Microbiol.

[6] European Centre for Disease Prevention and Control (Feb 14, 2024). Rapid risk assessment - Emergence of hypervirulent *Klebsiella pneumoniae* ST23 carrying carbapenemase genes in EU/EEA countries, first update. https://www.ecdc.europa.eu/en/publications-data/risk-assessment-emergence-hypervirulent-klebsiella-pneumoniae-eu-eea.

[7] Grundmann H, Glasner C, Albiger B (2017). Occurrence of carbapenemase-producing *Klebsiella pneumoniae* and *Escherichia coli* in the European survey of carbapenemase-producing Enterobacteriaceae (EuSCAPE): a prospective, multinational study. Lancet Infect Dis.

[8] Brolund A, Lagerqvist N, Byfors S (2019). Worsening epidemiological situation of carbapenemase-producing Enterobacteriaceae in Europe, assessment by national experts from 37 countries, July 2018. Euro Surveill.

[9] European Centre for Disease Prevention and Control (July 23, 2018). ECDC study protocol for genomic-based surveillance of carbapenem-resistant and/or colistin-resistant Enterobacteriaceae at the EU level. https://www.ecdc.europa.eu/en/publications-data/ecdc-study-protocol-genomic-based-surveillance-carbapenem-resistant-andor.

[10] European Centre for Disease Prevention and Control (Jan 14, 2019). Expert consensus protocol on carbapenem resistance detection and characterisation for the survey of carbapenem- and/or colistin-resistant Enterobacteriaceae. https://www.ecdc.europa.eu/en/publications-data/expert-consensus-protocol-carbapenem-resistance-detection-and-characterisation.

[11] European Centre for Disease Prevention and Control (Jan 14, 2019). Expert consensus protocol on colistin resistance detection and characterisation for the survey of carbapenem- and/or colistin-resistant Enterobacteriaceae. https://www.ecdc.europa.eu/en/publications-data/expert-consensus-protocol-colistin-resistance-detection-and-characterisation.

[12] European Committee on Antimicrobial Susceptibility Testing (EUCAST) (2019). Breakpoint tables for interpretation of MICs and zone diameters, version 9.0. https://www.eucast.org/fileadmin/eucast/pdf/Document_Archive/bacteria/breakpoint_tables/v_9.0_Breakpoint_Tables.xlsx.

[13] Bankevich A, Nurk S, Antipov D (2012). SPAdes: a new genome assembly algorithm and its applications to single-cell sequencing. J Comput Biol.

[14] Seemann T (2014). Prokka: rapid prokaryotic genome annotation. Bioinformatics.

[15] Lam MMC, Wick RR, Watts SC, Cerdeira LT, Wyres KL, Holt KE (2021). A genomic surveillance framework and genotyping tool for *Klebsiella pneumoniae* and its related species complex. Nat Commun.

[16] Tonkin-Hill G, MacAlasdair N, Ruis C (2020). Producing polished prokaryotic pangenomes with the Panaroo pipeline. Genome Biol.

[17] Katz LS, Griswold T, Morrison SS (2019). Mashtree: a rapid comparison of whole genome sequence files. J Open Source Softw.

[18] Kozlov AM, Darriba D, Flouri T, Morel B, Stamatakis A (2019). RAxML-NG: a fast, scalable and user-friendly tool for maximum likelihood phylogenetic inference. Bioinformatics.

[19] Argimón S, David S, Underwood A (2021). Rapid genomic characterization and global surveillance of *Klebsiella* using Pathogenwatch. Clin Infect Dis.

[20] Carattoli A, Zankari E, García-Fernández A (2014). In silico detection and typing of plasmids using PlasmidFinder and plasmid multilocus sequence typing. Antimicrob Agents Chemother.

[21] Argimón S, Abudahab K, Goater RJE (2016). Microreact: visualizing and sharing data for genomic epidemiology and phylogeography. Microb Genom.

[22] Magiorakos AP, Srinivasan A, Carey RB (2012). Multidrug-resistant, extensively drug-resistant and pandrug-resistant bacteria: an international expert proposal for interim standard definitions for acquired resistance. Clin Microbiol Infect.

[23] Peirano G, Chen L, Nobrega D (2022). Genomic epidemiology of global carbapenemase-producing *Escherichia coli,* 2015–2017. Emerg Infect Dis.

[24] Antimicrobial Resistance Collaborators (2022). Global burden of bacterial antimicrobial resistance in 2019: a systematic analysis. Lancet.

[25] Peirano G, Chen L, Kreiswirth BN, Pitout JDD (2020). Emerging antimicrobial-resistant high-risk *Klebsiella pneumoniae* clones ST307 and ST147. Antimicrob Agents Chemother.

[26] Wyres KL, Hawkey J, Hetland MAK (2019). Emergence and rapid global dissemination of CTX-M-15-associated *Klebsiella pneumoniae* strain ST307. J Antimicrob Chemother.

[27] Tryfinopoulou K, Linkevicius M, Pappa O (2023). Emergence and persistent spread of carbapenemase-producing *Klebsiella pneumoniae* high-risk clones in Greek hospitals, 2013 to 2022. Euro Surveill.

[28] Okomo U, Senghore M, Darboe S (2020). Investigation of sequential outbreaks of *Burkholderia cepacia* and multidrug-resistant extended spectrum β-lactamase producing *Klebsiella* species in a West African tertiary hospital neonatal unit: a retrospective genomic analysis. Lancet Microbe.

[29] Heinz E, Pearse O, Zuza A (2024). Longitudinal analysis within one hospital in sub-Saharan Africa over 20 years reveals repeated replacements of dominant clones of *Klebsiella pneumoniae* and stresses the importance to include temporal patterns for vaccine design considerations. Genome Med.

[30] Brennan C, DeLappe N, Cormican M (2022). A geographic cluster of healthcare-associated carbapenemase-producing hypervirulent *Klebsiella pneumoniae* sequence type 23. Eur J Clin Microbiol Infect Dis.

