# Supplementary material for: Persistent spread of carbapenemase-producing Klebsiella pneumoniae in acute care hospitals in 36 European countries (the CCRE survey): a prospective, multicentre, cross-sectional, epidemiological, microbiological, and genomic surveillance study
Source: Lancet Microbe. 2026 Jun;7(6):None. doi: 10.1016/j.lanmic.2025.101320 (PMC13259977; doi:10.1016/j.lanmic.2025.101320)
Supplement: Supplementary appendix 1 [file mmc1.pdf]

THE LANCET
Microbe

Supplementary appendix 1

This appendix formed part of the original submission and has been peer reviewed.
We post it as supplied by the authors.

Supplement to: Fröding I, David S, Yeats C, et al. Persistent spread of carbapenemase-producing *Klebsiella pneumoniae* in acute care hospitals in 36 European countries (the CCRE survey): a prospective, multicentre, cross-sectional, epidemiological, microbiological, and genomic surveillance study. *Lancet Microbe* 2026. https://doi.org/10.1016/j.lanmic.2025.101320

Table of Contents

Table A1 Susceptibility to antimicrobials other than carbapenems in *Klebsiella pneumoniae* SC isolates from the carbapenem- and/or colistin-resistant Enterobacterales (CCRE) survey 2

Figure A1 Flowchart of *Klebsiella pneumoniae* SC and *Escherichia coli** isolates in the carbapenem- and/or colistin-resistant Enterobacterales (CCRE) survey 3

Figure A2 Percentage of 1566 carbapenem-R/I and 1407 carbapenem-S *Klebsiella pneumoniae* SC isolates from the CCRE survey with phenotypic antimicrobial susceptibility testing results 4

Figure A3 Mash tree of 2973 *Klebsiella pneumoniae* SC isolates 5

Figure A4 Country distribution of dominant sequence types among 1566 carbapenem-R/I *Klebsiella pneumoniae* SC isolates from the CCRE survey 6

Figure A5 Carbapenemase gene distribution among the 1566 carbapenem-R/I *Klebsiella pneumoniae* SC isolates 7

Figure A6 Distribution of carbapenemase genes detected in 1566 carbapenem-R/I *Klebsiella pneumoniae* SC isolates from the CCRE survey, by country 8

Figure A7 A-B Proportion of A) sequence types and B) carbapenemase genes among carbapenem-R/I *Klebsiella pneumoniae* SC isolates from EuSCAPE (2013-14) and the CCRE survey (2019) 9

Table A1 Susceptibility to antimicrobials other than carbapenems in *Klebsiella pneumoniae* SC isolates from the carbapenem- and/or colistin-resistant Enterobacterales (CCRE) survey

Antimicrobial agent	Carbapenem-R/I *K. pneumoniae* SC				Carbapenem-R/I *K. pneumoniae* SC carrying carbapenemase genes				Carbapenem-S *K. pneumoniae* SC			
		R	I	S		R	I	S		R	I	S
	n tested	n (%, 95%CI)	n (%, 95%CI)	n (%, 95%CI)	n tested	n (%, 95%CI)	n (%, 95%CI)	n (%, 95%CI)	n tested	n (%, 95%CI)	n (%, 95%CI)	n (%, 95%CI)
Amoxicillin-clavulanic acid	1500	1490 (99·3, 98·6-99·7)	NA	10 (0·7, 0·3-1·4)	1351	1349 (99·9, 99·4-100·0)	NA	2 (0·1, 0·0-0·6)	1023	318 (31·1, 26·8-35·7)	NA	705 (68·9, 64·3-73·2)
Piperacillin-tazobactam	1516	1502 (99·1, 98·1-99·6)	6 (0·4, 0·2-1·0)	8 (0·5, 0·2-1·2)	1367	1365 (99·9, 99·4-100·0)	0	2 (0·1, 0·0-0·6)	1036	154 (14·9, 10·9-20·0)	108 (10·4, 9·1-11·9)	774 (74·7, 70·1-78·8)
Cefotaxime	1171	1103 (94·2, 88·6-97·1)	22 (1·9, 0·9-3·8)	46 (3·9, 1·9-7·9)	1028	970 (94·4, 88·0-97·4)	18 (1·8, 0·8-3·7)	40 (3·9, 1·7-8·4)	1037	282 (27·2, 19·1-37·1)	7 (0·7, 0·3-1·5)	748 (72·1, 62·7-80·0)
Ceftazidime	1520	1442 (94·9, 89·4-97·6)	20 (1·3, 0·6-3·1)	58 (3·8, 1·8-8·0)	1370	1297 (94·7, 88·3-97·7)	17 (1·2, 0·5-3·3)	56 (4·1, 1·8-8·9)	1064	282 (26·5, 18·3-36·7)	23 (2·2, 1·2-3·8)	759 (71·3, 62·4-78·9)
Cefepime	1457	1369 (94·0, 87·5-97·2)	23 (1·6, 0·7-3·5)	65 (4·5, 2·0-9·6)	1318	1241 (94·2, 87·2-97·4)	19 (1·4, 0·6-3·6)	58 (4·4, 1·9-10·0)	977	242 (24·8, 16·1-36·1)	25 (2·6, 1·3-5·0)	710 (72·7, 62·2-81·1)
Ceftazidime-avibactam	1126	277 (24·6, 16·1-35·6)	NA	849 (75·4, 64·4-83·9)	1017	267 (26·3, 16·5-39·1)	NA	750 (73·7, 60·9-83·5)	601	5 (0·8, 0·4-1·8)	NA	596 (99·2, 98·2-99·6)
Aztreonam	1007	888 (88·2, 82·7-92·1)	18 (1·8, 1·1-3·0)	101 (10·0, 6·8-14·6)	891	784 (88·0, 82·2-92·1)	12 (1·3, 0·6-2·8)	95 (10·7, 7·1-15·7)	850	204 (24·0, 16·2-34·0)	13 (1·5, 0·8-2·8)	633 (74·5, 65·2-81·9)
Ciprofloxacin	1484	1398 (94·2, 90·3-96·6)	11 (0·7, 0·4-1·6)	75 (5·1, 3·0-8·5)	1338	1274 (95·2, 91·8-97·2)	5 (0·4, 0·2-0·8)	59 (4·4, 2·5-7·6)	1015	271 (26·7, 21·4-32·8)	33 (3·3, 1·5-6·7)	711 (70·0, 63·7-75·7)
Trimethoprim-sulfamethoxazole	1404	1082 (77·1, 69·9-82·9)	18 (1·3, 0·7-2·2)	304 (21·7, 16·1-28·5)	1281	977 (76·3, 68·8-82·4)	17 (1·3, 0·7-2·4)	287 (22·4, 16·6-29·6)	922	306 (33·2, 24·4-43·4)	8 (0·9, 0·3-3·0)	608 (65·9, 56·8-74·0)
Gentamicin	1484	811 (54·6, 50·5-58·7)	18 (1·2, 0·4-3·3)	655 (44·1, 39·4-49·0)	1338	722 (54·0, 49·8-58·1)	17 (1·3, 0·4-3·7)	599 (44·8, 39·9-49·8)	1007	151 (15·0, 10·6-20·8)	14 (1·4, 0·2-7·6)	842 (83·6, 75·5-89·4)
Tobramycin	966	834 (86·3, 79·8-91·0)	9 (0·9, 0·5-1·8)	123 (12·7, 8·1-19·5)	867	755 (87·1, 79·9-91·9)	8 (0·9, 0·5-1·8)	104 (12·0, 7·2-19·4)	760	173 (22·8, 16·5-30·5)	16 (2·1, 0·5-8·4)	571 (75·1, 64·7-83·2)
Amikacin	1407	571 (40·6, 32·4-49·3)	140 (10·0, 4·7-19·8)	696 (49·5, 39·4-59·6)	1271	538 (42·3, 33·6-51·5)	127 (10·0, 4·6-20·5)	606 (47·7, 36·8-58·8)	943	35 (3·7, 2·1-6·4)	21 (2·2, 0·7-6·5)	887 (94·1, 89·0-96·9)
Colistin	1394	330 (23·7, 16·9-32·1)	NA	1064 (76·3, 67·9-83·1)	1264	312 (24·7, 17·6-33·4)	NA	952 (75·3, 66·6-82·4)	866	21 (2·4, 1·2-5·0)	NA	845 (97·6, 95·0-98·8)

R, resistant; I, susceptible, increased exposure; S, susceptible; NA, not available. 95%CI = 95% confidence interval

The table shows the number of tested isolates and number and proportion of tested isolates that were reported as resistant (R), susceptible, increased exposure (I) and susceptible (S) with 95% confidence intervals, to each antimicrobial agent for 1566 carbapenem-R/I K. pneumoniae SC isolates, 1398 K. pneumoniae SC isolates carrying carbapenemase genes and 1407 carbapenem-S K. pneumoniae SC isolates. Results are from antimicrobial susceptibility tests (AST), performed and interpreted by the NRLs, according to EUCAST breakpoint table v9.0, 2019. The number of isolates with AST results varies between antimicrobial agents. For some antimicrobials breakpoints have been changed in subsequent years. For piperacillin-tazobactam, isolates categorised as I in 2019, would be categorised as R from v11.0, 2021. For colistin, breakpoints are in brackets since the EUCAST breakpoint table v12.0, 2022. For isolates categorised as colistin S in the table, combination with another active agent or measure would be required. For aminoglycosides, breakpoints for monotherapy are limited to infections originating from the urinary tract, and isolates categorized as I for aminoglycosides in 2019, would be categorised as R since EUCAST breakpoint table v10.0, 2020. The proportion of isolates that were tested for tobramycin was <60%, for this reason these results are considered uncertain, and the results were not included in Figure 1.

Figure A1 Flowchart of *Klebsiella pneumoniae* SC and *Escherichia coli isolates in the carbapenem- and/or colistin-resistant Enterobacterales (CCRE) survey**

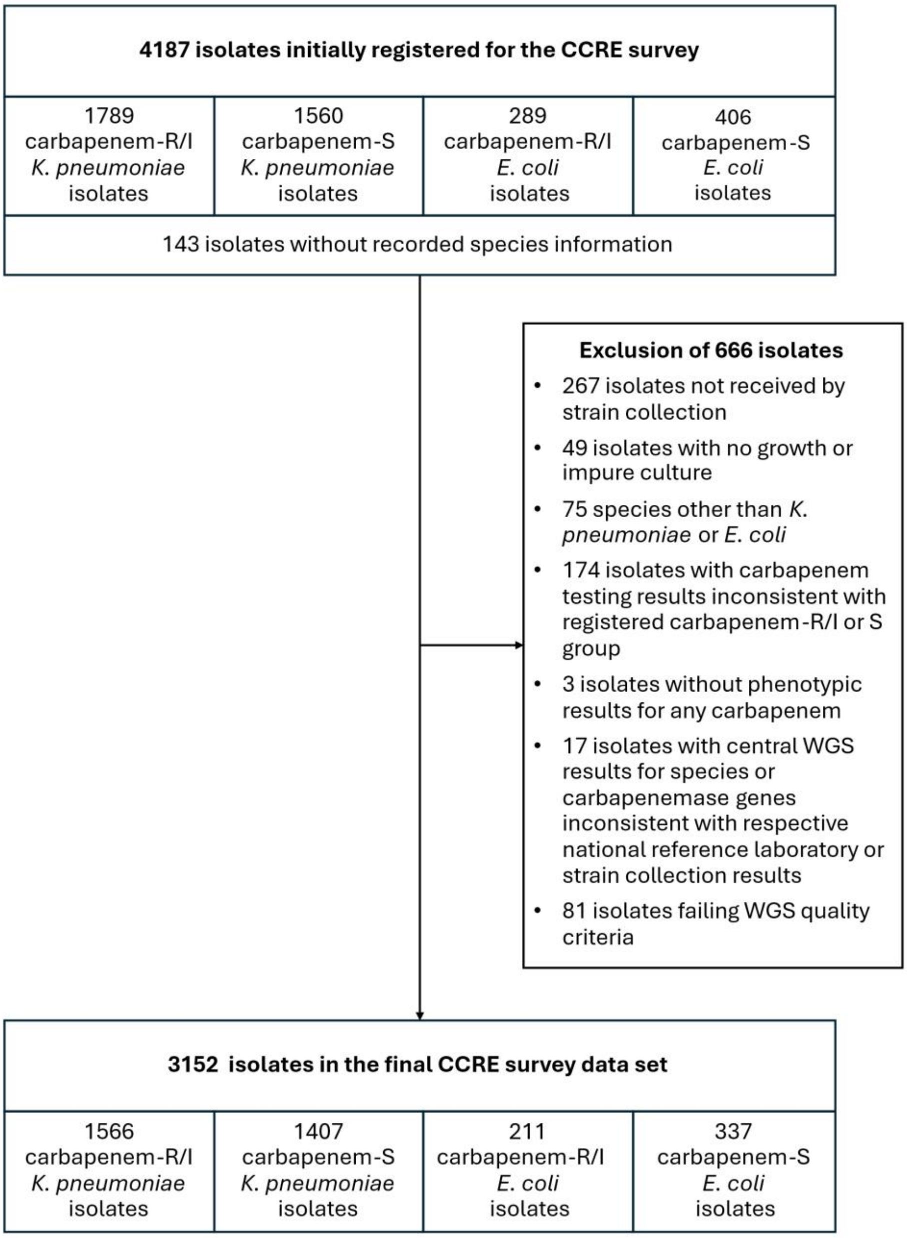

**The analysis of the* E. coli *isolates is described in a separate manuscript*.

CCRE survey, survey of carbapenem-and/or colistin-resistant Enterobacterales; R, resistant; I, susceptible, increased exposure; S, susceptible; WGS, whole genome sequencing.

Figure A2 Percentage of 1566 carbapenem-R/I and 1407 carbapenem-S *Klebsiella pneumoniae* SC isolates from the CCRE survey with phenotypic antimicrobial susceptibility testing results

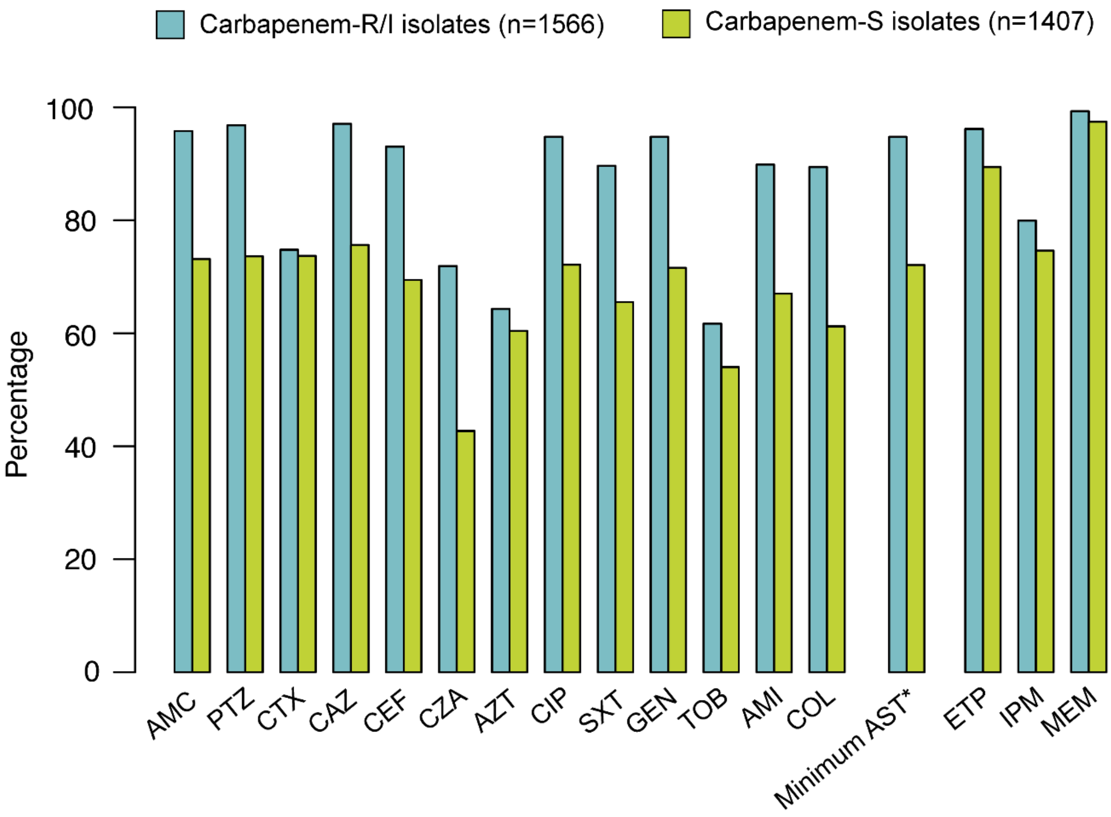

R, resistant; I, susceptible, increased exposure; S, susceptible.
*Abbreviations: AMC – amoxicillin-clavulanic acid, PTZ – piperacillin-tazobactam, CTX – cefotaxime, CAZ – ceftazidime, CEF – cefepime, CZA – ceftazidime-avibactam, AZT – aztreonam, CIP – ciprofloxacin, SXT – trimethoprim-sulfamethoxazole, GEN – gentamicin, TOB–tobramycin, AMI – amikacin, COL – colistin, ETP – ertapenem, IPM – imipenem, MEM – meropenem. *Minimum antimicrobial susceptibility testing (AST) is defined as AST performed for ≥1 carbapenem, ≥1 extended-spectrum cephalosporin and ≥1 fluoroquinolone.*

Figure A3 Mash tree of 2973 *Klebsiella pneumoniae* SC isolates

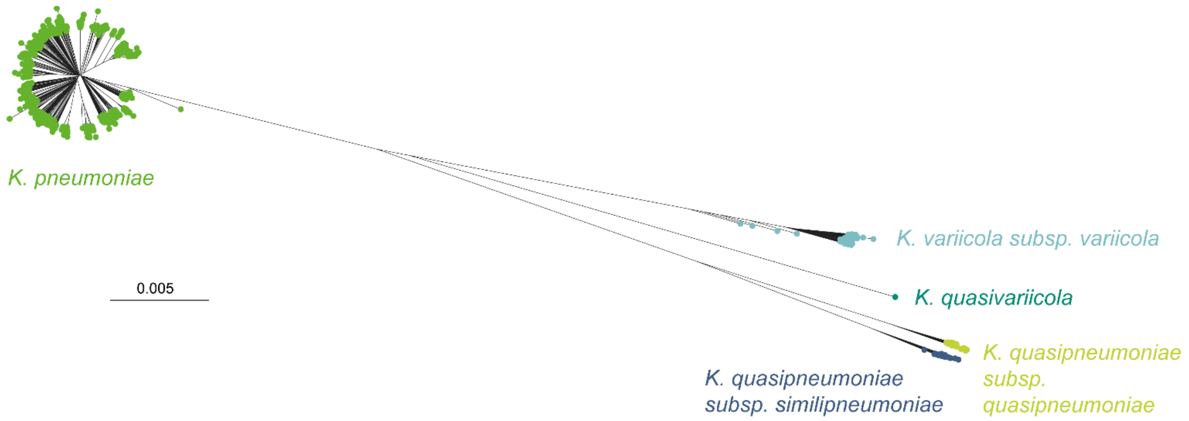

Isolates are coloured by species/ subspecies. The scale bar represents the Mash distance between isolates. An interactive version of the tree with additional metadata and genotyping data is available at: ***https://microreact.org/project/klebsiella-ccre-survey***

Figure A4 Country distribution of dominant sequence types among 1566 carbapenem-R/I *Klebsiella pneumoniae* SC isolates from the CCRE survey

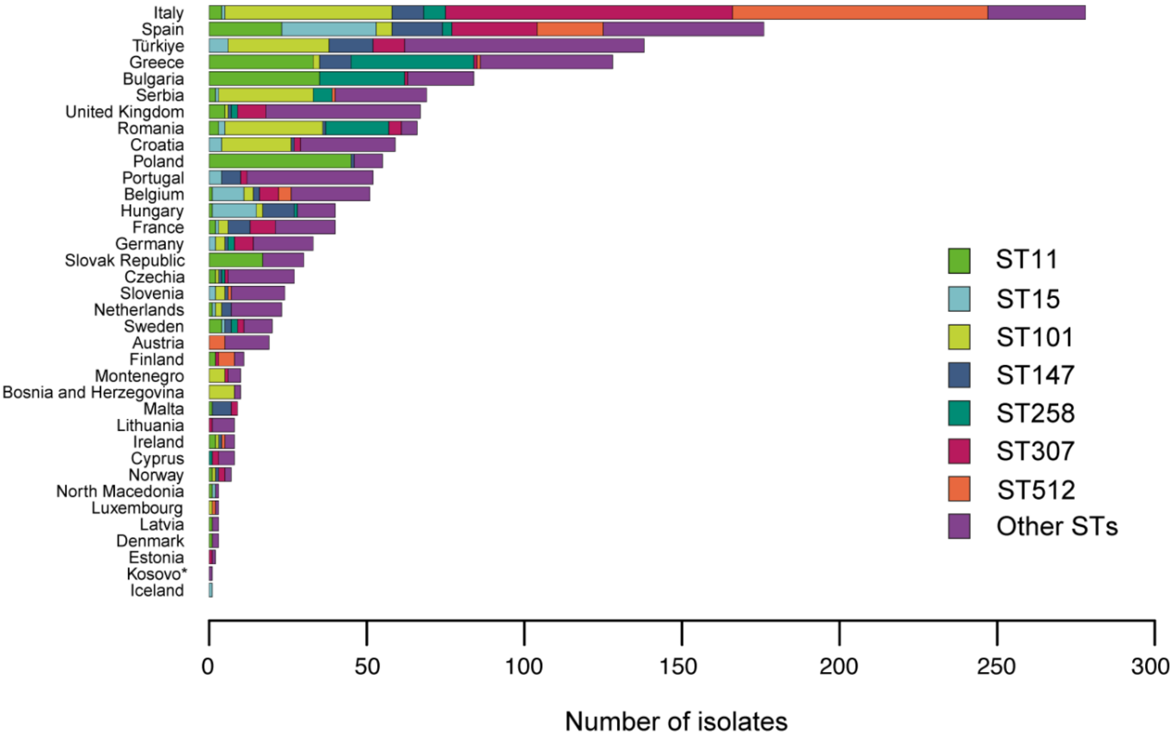

**This designation is without prejudice to positions on status and is in line with United Nations Security Council Resolution 1244/99 and the International Court of Justice Opinion on the Kosovo declaration of independence.*
R, resistant; I, susceptible, increased exposure.

Figure A5 Carbapenemase gene distribution among the 1566 carbapenem-R/I *Klebsiella pneumoniae* SC isolates

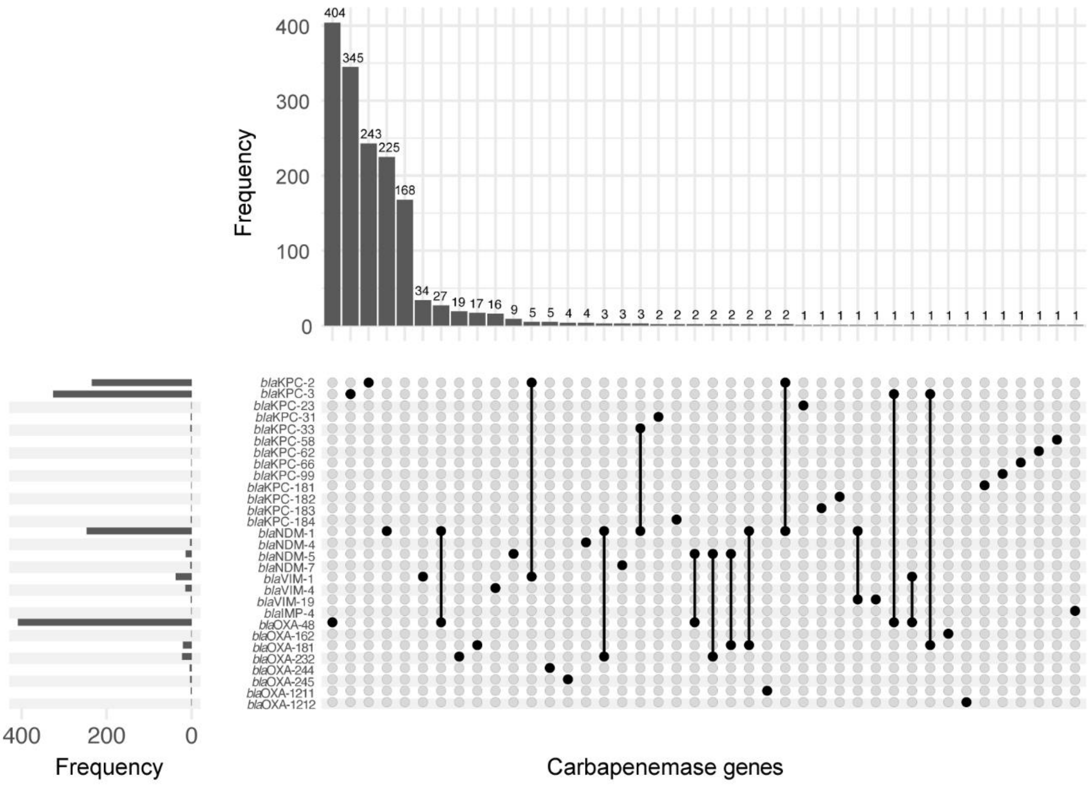

R, resistant; I, susceptible, increased exposure.
The frequencies of all the combinations identified are shown in the upper bar plot, while the frequencies of individual genes are shown on the left. Six variants with high similarity to known carbapenemase genes were newly detected in this study (bla$_{KPC\text{-}181}$*, bla*$_{KPC\text{-}182}$*, bla*$_{KPC\text{-}183}$*, bla*$_{KPC\text{-}184}$*, bla*$_{OXA\text{-}1211}$*, bla*$_{OXA\text{-}1212}$*).*

Figure A6 Distribution of carbapenemase genes detected in 1566 carbapenem-R/I *Klebsiella pneumoniae* SC isolates from the CCRE survey, by country

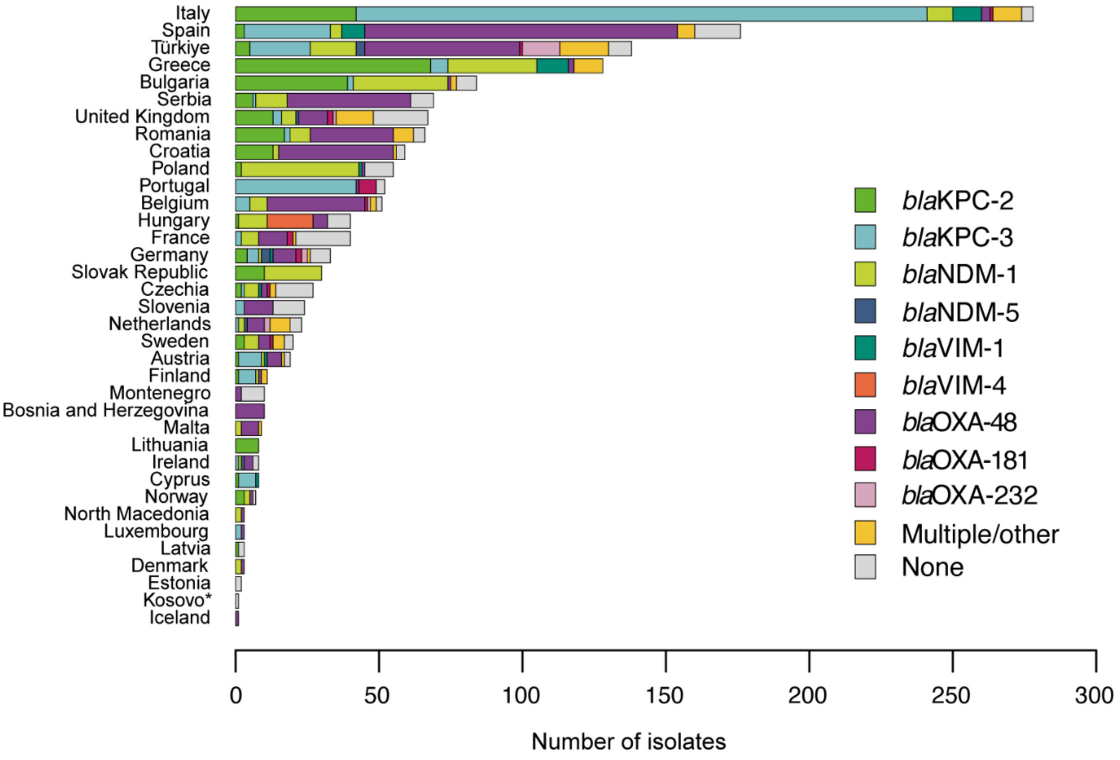

**This designation is without prejudice to positions on status and is in line with United Nations Security Council Resolution 1244/99 and the International Court of Justice Opinion on the Kosovo declaration of independence.*
R, resistant; I, susceptible, increased exposure.
Genes comprising ≤1% of all carbapenemase genes are included in the "Multiple/other" category.

Figure A7 A-B Proportion of A) sequence types and B) carbapenemase genes among carbapenem-R/I *Klebsiella pneumoniae* SC isolates from EuSCAPE (2013-14) and the CCRE survey (2019)

A.

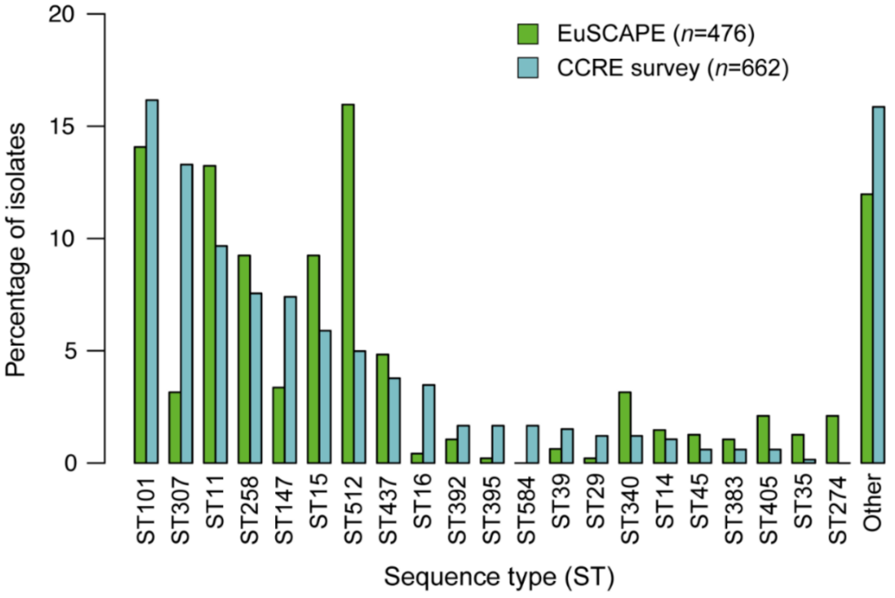

Only isolates from 113 hospitals that contributed K. pneumoniae *SC isolates to both data sets were included. Sequence types making up ≥1% of carbapenem-R/I isolates in either of the sample sets are shown.*

B.

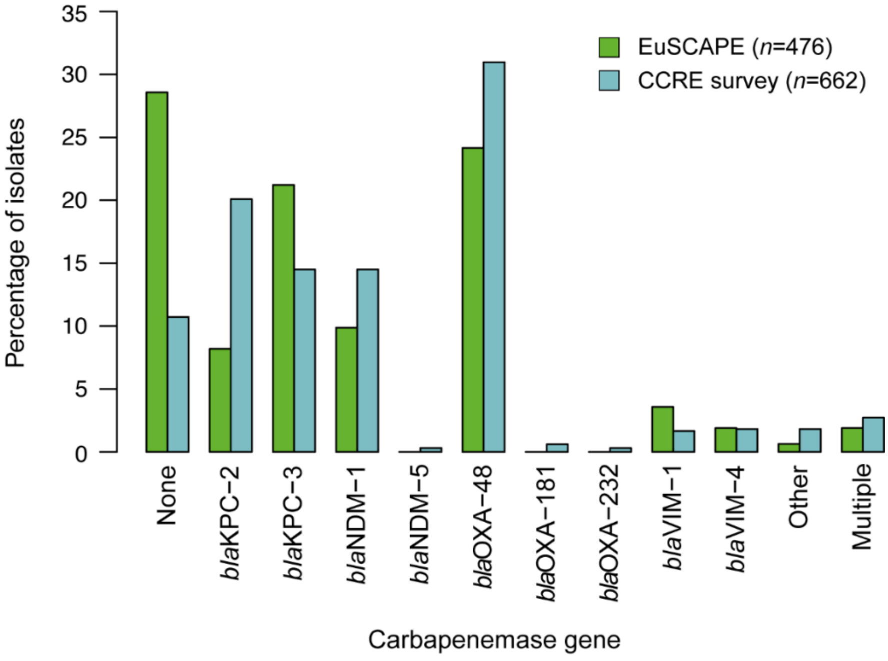

R, resistant; I, susceptible, increased exposure.
Only isolates from 113 hospitals that contributed K. pneumoniae *SC isolates to both data sets were included. Genes comprising ≥1% of carbapenemase genes in the CCRE survey isolates are shown.*
